# Effects of coarse corn or oat hulls on growth performance, intestinal health, and microbiota modulation in underperforming broilers

**DOI:** 10.1016/j.aninu.2025.04.012

**Published:** 2025-08-06

**Authors:** Muhammad Zeeshan Akram, Ester Arévalo Sureda, Matthias Corion, Luke Comer, Haoran Zhao, Martine Schroyen, Nadia Everaert

**Affiliations:** a Nutrition and Animal-Microbiota Ecosystems Laboratory, Department of Biosystems, KU Leuven, Heverlee 3000, Belgium; b Precision Livestock and Nutrition Unit, Gembloux Agro-Bio Tech, University of Liège, Gembloux, Belgium

**Keywords:** Coarse corn, Oat hulls, Underperforming broiler, Gut health, Microbiota

## Abstract

Intra-flock body weight (BW) variability in broilers increases production costs, as underperforming chicks often show suboptimal gut development and performance. Increasing grain particle size and dietary fiber content has been shown to improve digestive efficiency and intestinal health. This study investigated whether dietary inclusion of coarse corn (CC) and oat hulls (OH) could improve gut health and reduce the performance gap between low- and high-BW (LBW and HBW) broilers. On d 7, 1400 Ross 308 male broilers were categorized as LBW or HBW, with 504 LBW chicks assigned to 4 isocaloric and isonitrogenous diets with 10% fine corn (LBWC), 7% CC with 3% fine corn (LBW + CC), 3% OH with 10% fine corn (LBW + OH), or 7% CC and 3% OH (LBW + CO). High BW chicks received a 10% fine corn diet (HBWC). Each group had 6 replicates with 21 chicks per pen. The HBWC group showed the highest BW at each timepoint (*P* < 0.05). By d 38, LBW + OH chicks had significantly reduced the weight difference with HBWC chicks and significantly outperformed LBWC chicks (*P* < 0.001), whereas other groups showed intermediate values. Coarse corn and OH, individually or combined, reduced the relative plasma FITC-dextrann concentration d 14 (*P* = 0.014) and increased gizzard weights on d 21 and 38 (*P* < 0.05) as compared with LBWC group. The LBW + OH group showed increased pancreas relative weight on d 21 (*P* = 0.005, vs. HBWC) and villus height (*P* = 0.042, vs. LBWC) on d 38. Additionally, LBW + OH group reduced isobutyrate and isovalerate levels in cecum (*P* < 0.05, vs. HBWC and LBWC) on d 21, and upregulated ileal genes related to gut barrier function (*CLDN1,* vs. HBWC and LBWC; *CLDN4*, vs. HBWC; *CLDN5,* vs. LBWC), amino acid and glucose transporters (*SLC15A1* and *SLC1A4,* vs. HBWC and LBWC), and immune function (*NOS2*, vs. HBWC and LBWC; *TLR4*, vs. HBWC) on d 14 (*P* < 0.05), and sodium-phosphate transporter *SLC34A2* (*P* = 0.049, vs. HBWC) on d 38. LBW + CC birds upregulated *SLC15A1* (vs. HBWC and LBWC) on d 38 (*P* < 0.001). *Lactobacillus* was enriched in the cecum of HBWC birds, while *Escherichia-Shigella* was abundant in LBWC birds on d 14, with CC and OH promoting beneficial bacterial shifts in LBW groups. Overall, incorporating structural components into diets, particularly 3% OH, enhanced gastrointestinal development, intestinal integrity, and growth performance in LBW broilers. These improvements reduced disparities in BW between LBW and HBW birds, thereby contributing to more uniform flock performance at slaughter age.

## 1 Introduction

The broiler industry confronts persistent challenges in managing weight heterogeneity, particularly for low body weight (LBW) broilers that consistently underperform compared to their normal-weight counterparts. These birds represent a significant economic and production challenge, characterized by suboptimal performance, health issues and welfare concerns (Hughes et al., 2017). Low body weight broilers are uniquely vulnerable, exhibiting profound physiological limitations that extend beyond mere growth restrictions. These limitations include severe gastrointestinal tract (GIT) developmental impairments, such as increased intestinal permeability and the downregulation of gut barrier function proteins and nutrient transporters (Akram et al., 2024c; Zhang et al., 2022). Furthermore, LBW broilers often harbor dysbiotic microbiota, leading to elevated proinflammatory cytokine levels and increased intestinal inflammation (Akram et al., 2024a,b,c; Zhang et al., 2022), which further compromises their growth and health.

Optimal broiler growth and production fundamentally depend on efficient nutrient digestion and absorption, which are intrinsically linked to a well-functioning digestive system (El Sabry and Yalcin, 2023). In recent years, nutritional strategies aiming to modulate gut structure and function have gained prominence, particularly the incorporation of dietary insoluble fiber sources and coarse feed particles (Jiménez-Moreno et al., 2013; Xu et al., 2015). Adding insoluble fiber sources such as oat hulls (OH) has been found to improve nutrient retention, digestion, and growth in broilers (Naeem et al., 2023). Likewise, the addition of coarse corn (CC) to pelleted diets enhances protein digestibility, energy utilization, live performance, and litter quality (Xu et al., 2015). These benefits are attributed to the physical properties of these feed ingredients, which stimulate gizzard development (Sarbast K Kheravii et al., 2017), and increase pancreatic enzyme secretion, such as amylase and chymotrypsin, driven by gizzard activity. Furthermore, a well-developed gizzard promotes the release of cholecystokinin (CCK), which stimulates reverse peristalsis, prolonging digesta transit time and enabling more thorough digestion (Denbow, 2015; Svihus et al., 2004; Svihus, 2011). Consequently, slower digesta transit increases nutrient digestion and absorption by maximizing the contact time with absorptive cells (Washburn, 1991). Besides the chemical composition and particle size, the physical structure of feed including pellet size and hardness may influence digestive development. Harder pellets can further stimulate gizzard activity by resisting breakdown, thereby enhancing the mechanical stimulation of the digestive tract (Abdollahi and Ravindran, 2013). While the breakdown of fiber in poultry is minimal in terms of energy provision, it may still influence the nutritional value of feed through interactions with other nutrients. Unlike soluble fiber, which can hinder nutrient digestion and absorption due to increased digesta viscosity (Smits et al., 1997), insoluble fiber supports chicken growth by improving the nutrient digestibility in the upper GIT and stimulating microbial fermentation in the lower tract (Naeem et al., 2023). However, the effects of insoluble fiber can be context-dependent. At inappropriate inclusion levels or depending on the fiber source and bird physiology, insoluble fiber may impair nutrient utilization or lead to undesirable outcomes such as wet litter or sticky droppings, often considered antinutritional effects in broiler production systems (Jha and Mishra, 2021). The physical attributes of OH and CC may also exert microbiota-modulating effects. The lignin-rich matrix of fiber materials just as in OH can act as a fermentable substrate for beneficial microbes (Kheravii et al., 2017), producing short-chain fatty acids (SCFAs) with anti-inflammatory and trophic effects on the gut epithelium. Similarly, the structural complexity of CC may support the proliferation of specific bacterial taxa that favor gut health and metabolic efficiency (Yan et al., 2022). While individual studies have explored the effects of CC and OH on nutrient digestibility and growth performance, their impact on gut microbiota and intestinal health is relatively underexplored.

The positive effects of coarse grain particles and insoluble fiber on broiler performance are well documented (Sarbast K Kheravii et al., 2017; Kheravii et al., 2018a). However, their potential benefits in LBW broilers, which are characterized by impaired growth performance and physiological development, remain elusive. Given the unique physiological deficits in LBW birds, such interventions may offer greater relative benefits by compensating for early developmental delays. Therefore, this study aimed to evaluate the individual and combined effects of dietary CC and OH on growth performance, GIT morphology, intestinal health markers, and microbiota characteristics in LBW broilers. We hypothesized that the inclusion of these structural dietary components would stimulate gizzard activity, improve gut function and microbial balance, thereby enhancing growth and narrowing the performance disparity between LBW and high body weight (HBW) broilers.

## 2 Materials and methods

### 2.1 Animal ethics statement

The study was performed at TRANSfarm, KU Leuven, Bierbeek, Belgium, following approval from the KU Leuven Ethical Committee for Animal Experimentation under project number 112/2023.

### 2.2 Experimental diets

Isocaloric and isonitrogenous wheat-based broiler diets were formulated to meet nutritional requirements across different growth phases (Table 1). All broilers received a crumbled-form pre-starter diet in the first week. Thereafter, 4 pelleted experimental diets were formulated in a commercial feed mill (Vanden Avenne Commodities, Ooigem, Belgium) using conditioning with expander: a commercial broiler diet with 10% finely ground corn (control), a diet formulated with 7% CC and 3% finely ground corn (CC), a diet containing 10% finely ground corn and 3% OH (OH), and a combination diet with 7% CC and 3% OH (CO). The grower diets were fed until d 16, followed by the finisher diets provided until the end of the trial.

**Table 1:** Composition and nutrient level of the wheat-based diet (%, DM basis).

Item	Pre-starter (d 1 to 7)	Grower (d 8 to16)				Finisher (d 17 to 38)			
		Control	CC	OH	CO	Control	CC	OH	CO
**Ingredients**									
Wheat	43.48	52.93	52.92	49.35	49.35	55.89	55.89	53.47	53.47
Soyabean meal	28.21	26.16	26.17	28.48	28.46	21.84	21.84	24.69	24.67
Coarse corn	0.00	0.00	7.00	0.00	7.00	0.00	7.00	0.00	7.00
Oat hull	0.00	0.00	0.00	3.00	3.00	0.00	0.00	3.00	3.00
Fine corn	15.00	10.00	3.00	10.00	3.00	10.00	3.00	10.00	3.00
Soya oil	4.77	4.83	4.83	5.49	5.49	4.83	4.83	5.08	5.08
Sunflower meal	3.57	2.40	2.40	0.00	0.00	3.90	3.90	0.00	0.00
Monocalcium phosphate	1.29	0.89	0.89	0.92	0.92	0.93	0.93	0.97	0.97
Limestone	1.26	0.93	0.93	0.92	0.92	0.84	0.84	0.82	0.82
Salt	0.20	0.22	0.22	0.22	0.22	0.21	0.21	0.22	0.22
Na-bicarbonate	0.20	0.17	0.17	0.16	0.16	0.18	0.18	0.17	0.17
Choline 75%	0.09	0.09	0.09	0.09	0.09	0.09	0.09	0.09	0.09
Lysine	0.66	0.48	0.48	0.47	0.49	0.50	0.50	0.49	0.50
Methionine	0.36	0.29	0.29	0.30	0.30	0.26	0.26	0.28	0.28
L-Valine	0.12	0.06	0.06	0.06	0.06	0.05	0.05	0.06	0.06
L-Isoleucine	0.07	0.02	0.02	0.02	0.02	0.02	0.02	0.02	0.02
L-Arginine	0.06	0.00	0.00	0.00	0.00	0.00	0.00	0.02	0.03
L-Threonine	0.21	0.15	0.15	0.14	0.14	0.14	0.14	0.30	0.30
Vitamin E	0.01	0.01	0.01	0.01	0.01	0.01	0.01	0.01	0.01
Vitamin premix^1^	0.39	0.31	0.31	0.31	0.31	0.30	0.30	0.30	0.30
Decoquinate 2	0.05	0.05	0.05	0.05	0.05	0.00	0.00	0.00	0.00
Phytase^3^	0.008	0.008	0.008	0.008	0.008	0.008	0.008	0.008	0.008
Xylanase^4^	0.00	0.01	0.01	0.01	0.01	0.01	0.01	0.01	0.01
**Nutrient levels**^5^									
Metabolizable energy, kcal/kg	2975	2925	2925	2925	2925	2950	2950	2950	2950
Crude protein	21.30	20.20	20.18	20.17	20.21	19.00	19.09	19.02	19.04
Crude fat	5.92	5.23	5.46	5.82	5.93	5.66	5.68	6.08	5.84
Crude fiber	3.18	3.07	3.06	3.58	3.57	3.22	3.21	3.56	3.55
NDF	8.37	9.32	9.11	17.7	15.46	9.01	8.45	12.83	11.38
ADF	4.61	4.58	4.83	5.01	5.41	4.37	4.21	4.95	4.49
ADL	0.63	0.62	0.62	0.70	0.68	0.69	0.67	0.71	0.73
Digestible lysine	1.22	1.08	1.05	1.10	1.08	1.00	1.01	1.04	1.01
Calcium	0.7	0.69	0.69	0.72	0.73	0.69	0.73	0.69	0.68
Phosphorus	0.70	0.57	0.51	0.59	0.56	0.59	0.60	0.57	0.57
Sodium	0.14	0.13	0.13	0.14	0.14	0.15	0.14	0.14	0.14
Chloride	0.20	0.21	0.19	0.22	0.20	0.19	0.21	0.23	0.20
Potassium	0.94	0.90	0.88	0.94	0.93	0.85	0.85	0.87	0.88
Magnesium	0.21	0.20	0.21	0.18	0.19	0.18	0.17	0.19	0.18

Control = a commercial broiler diet with 10% finely ground corn; CC = a diet formulated with 7% coarse corn and 3% finely ground corn; OH = a diet containing 10% finely ground corn and 3% oat hulls; CO = a diet containing 7% coarse corn and 3% oat hulls.

1 Premix provided per kilogram of diets: vitamin A 10,000 IU, vitamin D_3_ 2750 IU, 25-hydroxycho2ecalciferol 0.056 mg, vitamin E 90 mg, copper 15 mg, iron 15 mg, manganese 85 mg, zinc 50 mg, iodine 2 mg, and selenium 0.4 mg.

2 Provided per kilogram of diets: 30.3 mg of decoquinate.

3 Provided per kilogram of diets: 500 FTU.

4 Provided per kilogram of diets: 10 IU.

5 Metabolizable energy was calculated, while all other nutrient levels were analyzed.

Fine corn, wheat, and soybean meal were ground using a hammer mill fitted with a 4-mm sieve, while coarse corn was processed using a roller mill with sequential gap settings of 1.8, 1.6, and 1.5 mm. Oat hulls, initially pelleted, were reground using a roller mill with fixed gaps of 3.6 mm. All experimental diets were pelleted using a die with 3.2-mm holes and a roll-die distance of 0.2 mm. Pelleting involved an expander time of approximately 5 s at 20 bar (2.0 MPa) and 80 °C, followed by a conditioning phase at 65 °C for 10 s with 2.1% steam addition and 12% initial feed moisture.

The particle size distribution of the OH was assessed through dry sieving in duplicate. Results indicated that 62% of particles were greater than 4.00 mm, 10% between 3.15 and 4.00 mm, 13% between 2.00 and 3.15 mm, 11% between 1.00 and 2.00 mm, and 4% < 1.00 mm. The particle size distribution (%), geometric mean diameter (GMD) and geometric standard deviation (GSD) of the experimental feeds were determined through wet sieving in duplicate (Table 2). A 20-g feed sample was soaked in 400 mL of distilled water for 1 h at room temperature. The sample was then sieved using a vibratory sieve shaker (Retch NV, Aartselaar, Belgium) equipped with sieves with mesh sizes of 2000, 1000, 500, 200, 90, 50, and 38 μm. The feed and water suspension were deposited onto the top sieve, and sieving was performed for 10 min with a water flow rate of 2.0 to 2.3 L/min, followed by 1 min without water flow to drain excess moisture. The fractions retained on each sieve were collected separately in Falcon tubes, freeze-dried, and stored in a desiccator until weighing. The average particle size (*d*_av_) was calculated according to the equation:

$$d_{\text{av}}=\sum d_{i}\times m_{i}\text{,}$$

where *d*_*i*_ represents the mesh size of sieve *i*, and *m*_*i*_ represents the mass percentage of the fraction retained on sieve *i*.

**Table 2:** Wet sieving particle size distribution (%) and geometric mean diameter (GMD) of experimental diets used in pre-starter, grower and finisher phases for low- and high-weight broilers.

Item	Pre-starter (d 1 to 7)	Grower (d 8 to 16)				Finisher (d 17 to 38)			
		Control	CC	OH	CO	Control	CC	OH	CO
**Sieve size**									
2.000 mm	1.3	1.0	10.1	5.4	7.4	4.7	13.7	5.4	15.5
1.000 mm	6.1	7.6	16.8	8.4	14.1	9.4	18.9	11.4	25.1
0.500 mm	10.2	9.1	12.1	13.1	12.7	13.0	12.2	11.7	10.0
0.200 mm	12.7	11.0	8.4	9.8	10.6	9.7	7.6	8.7	6.3
0.090 mm	10.2	9.4	5.1	8.4	5.3	8.4	4.6	7.5	3.8
0.050 mm	10.2	7.2	6.1	6.4	6.4	6.4	5.5	5.7	4.5
0.038 mm	6.3	9.4	3.0	8.4	3.2	8.4	2.7	7.5	2.3
<0.038 mm	43.1	45.3	38.4	40.2	40.3	40.1	34.7	42.0	32.6
>1.000 mm	7.4	8.6	26.9	14.0	21.5	14.1	32.6	16.8	40.6
**GMD ± GSD**	360 ± 54.1	372 ± 48.7	582 ± 57.6	416 ± 51.5	532 ± 82.2	413 ± 45.9	621 ± 108.2	458 ± 53.3	698 ± 101.2

Control = a commercial broiler diet with 10% finely ground corn; CC = a diet formulated with 7% CC and 3% finely ground corn; OH = a diet containing 10% finely ground corn and 3% OH; CO = a diet containing 7% CC and 3% OH; GSD = geometric standard deviation.

Except stated otherwise, all chemical analyses were done using AOAC (2016) methods. Crude protein was analyzed by the Kjeldahl method (method 990.03), and crude fat was via Soxhlet extraction (method 920.39). Crude fiber was analyzed using the fiber bag technique (method 978.10). Neutral detergent fiber (NDF) and acid detergent fiber (ADF) were analyzed following the procedures described by Van Soest et al. (1991), while acid detergent lignin (ADL) was measured following method 973.18. Digestible lysine levels were calculated based on the analyzed amino acid profile, analyzed using high-performance liquid chromatography following method 982.30. Calcium, phosphorus, sodium, potassium, and magnesium were quantified using inductively coupled plasma optical emission spectrometry following method 985.01. Chloride content was analyzed using potentiometric titration following method 943.01.

### 2.3 Animals, husbandry, and data collection

A total of 1400 one-day-old male Ross 308 broiler chicks (initial BW: 44.5 ± 3.21 g) were obtained from a commercial hatchery (Belgabroed NV, Aartselaar, Belgium). All chicks were fed a pre-starter diet until d 7, after which individual BW was recorded, and the birds were categorized into low-, medium-, and high-weight groups on the basis of BW distribution. Day 7 BW is a strong predictor of final BW, and selection at this age enables biologically relevant classification of LBW and HBW phenotypes before compensatory growth mechanisms begin to manifest, which typically occur during later stages of development (Akram et al., 2024c,b). The chicks on the lower end of the BW range were designated as LBW (*n* = 504), and further divided into 4 treatment groups: LBWC, LBW + CC, LBW + OH, and LBW + CO, each comprising 126 chicks receiving either the control diet or one of the experimental diets (CC, OH, or CO). Additionally, the birds on the higher end of the BW (HBWC, *n* = 126) received the control diet with fine corn, whereas the remaining medium BW chicks (*n* = 770) were excluded from the experiment. The experiment consisted of 5 groups with 6 replicate pens per group (1.3 m^2^ per pen), with each pen containing 21 birds. Pen floors were covered with 3 cm of wood shavings. Feed and water were provided ad libitum throughout the study. The light schedule started with 1 h of darkness on d 1, increasing by 1 h per day to 6 h of darkness, which was maintained thereafter. The initial temperature was set at 33 °C and decreased by 0.5 °C daily until it reached 21.5 °C on d 21, after which it remained constant.

### 2.4 Sampling and measurements

BW was individually recorded, and feed intake was recorded per pen after each dietary phase. The average daily gain (ADG), mortality-corrected average daily feed intake (ADFI) and feed conversion ratio (FCR) were calculated for the grower and finisher phases. The birds weighing nearest to the pen’s average weight (*n* = 12) were sacrificed through electrical stunning followed by decapitation on d 14, 21, and 38. Dissection was performed, and the weights of the gizzard, liver, pancreas, small intestine, and cecum were determined. The small intestine and cecum weights were measured without emptying the digesta, and their length was also recorded. The relative organ weights were expressed as g/100 g BW, and the relative lengths of the small intestine and cecum were calculated as cm/100 g BW. Digesta samples from both caeca were collected, placed in 2-mL vials, snap-frozen in liquid nitrogen, and stored at −80 °C for microbiota and volatile fatty acid (VFA) analysis. Ileum sections from the midpoint were taken for histomorphological examination on d 14, 21 and 38, while ileal tissue samples were snap-frozen and stored at −80 °C for high-throughput qPCR gene expression analysis on d 14 and 38. Two chickens per pen in each group were randomly selected on d 14, 21 and 38 for intestinal permeability tests via fluorescein isothiocyanate (FITC)-dextran (4000 kDa; Sigma–Aldrich, St. Louis, MO, USA).

### 2.5 Ileal histomorphology

Ileum samples were fixed in 4% formaldehyde for 48 h and afterwards stored in 70% ethanol. Histology sections were embedded in paraffin, sectioned, and stained with alcian blue-periodic acid–Schiff according to the standard procedure of the GIGA immunohistochemistry platform (ULiège, Belgium). The microscopy images were analyzed via NDP.view2 software (Hamamatsu Photonics K.K., Hamamatsu, Japan). Villus height (VH) and crypt depth (CD) were measured for 20-well-oriented villus-crypt units per bird, and the VH/CD ratio was calculated.

### 2.6 Intestinal permeability

FITC-dextran solution (2.2 mg/mL per bird) was administered orally, and blood samples (1 mL) were collected from the jugular vein 2.5 h post gavage. Blood samples were centrifuged at 4 °C at 3000× *g*. A standard series was created, and plasma samples diluted in phosphate-buffered saline (1:5) were analyzed in duplicate via a 96-well microplate reader (CLARIOstar Plus, BMG LABTECH GmbH, Offenburg, Germany) with an excitation wavelength of 485 nm and an emission wavelength of 530 nm. Plasma FITC-dextran concentrations (ng/mL) were calculated using a standard curve. The relative concentration of FITC-dextran was calculated as ng/mL per 100 g BW.

### 2.7 Cecal volatile fatty acid analysis

Short-chain fatty acids (acetate, propionate, butyrate, valerate, and caproate) and branched-chain fatty acids (BCFAs: isobutyrate, isovalerate, and isocaproate) were measured according to a modified method from previous study (Van Craeyveld et al., 2008). Briefly, approximately 250 mg of cecal content was weighed into 2-mL Eppendorf tubes, placed on ice, and mixed with 50 μL of MHA-2 internal standard solution and 80 μL of 6 mol/L HCl. The samples were vortexed and were left on ice for 20 min and then mixed with 25% NaCl and tertiary-butyl methyl ether. After centrifugation at 4 °C (10,000 × *g* for 5 min), 600 μL of the supernatant was transferred to a 1.5-mL Eppendorf tube containing anhydrous sodium sulfate, vortexed, and centrifuged again. A 200-μL aliquot was pipetted into screw-neck vials with conical glass inserts and stored at −20 °C until analysis. Volatile fatty acids were quantified via an HP 6890 Series GC System equipped with an automatic liquid sampler, flame ionization detector, and DB-FFAP capillary column (30 m length, 0.32 mm internal diameter, 0.25 μm film thickness; Agilent Technologies, Santa Clara, CA, USA). The carrier gas was nitrogen at a flow rate of 25 mL/min, with the column at 130 °C and the injector and detector at 195 °C. SCFA and BCFA concentrations were calculated in mmol/g wet digesta on the basis of calibration curves.

### 2.8 Gene expression through high-throughput qPCR

#### 2.8.1 Selection of genes, primer design and validation

A total of 92 genes (13 housekeeping genes and 79 target genes) involved in intestinal barrier function, nutrient transport, immune response, metabolism, and oxidative homeostasis were selected based on published literature (Akram et al., 2024c, 2025). Details on the genes and their primary functions are provided in Table S1.

Primers were designed using the NCBI Primer-Blast tool to span exon–exon junctions to minimize genomic DNA amplification. Specificity was confirmed by melting curve analysis following qPCR amplification, which revealed single peaks for all primers, indicating that there was no non-specific amplification or primer-dimer formation. Verification using agarose gel electrophoresis revealed single and distinct bands at the expected molecular weights for each amplicon. The primer efficiency was optimized between 90% and 110%, with *R*^2^ values exceeding 0.99, using 3-fold serial dilutions of pooled cDNA derived from all samples on a QuantStudio 6 Real-Time PCR System (Thermo Fisher Scientific, Waltham, MA, USA).

#### 2.8.2 RNA extraction

Total RNA was extracted from ileal tissue samples using the ReliaPrep RNA Miniprep System (Promega Corporation, Madison, WI, USA) according to the manufacturer’s protocol. The RNA concentration and purity were determined by spectrophotometry (Nanodrop 2000, Thermo Fisher Scientific, Waltham, MA, USA), whereas the integrity of the RNA was verified on 1% agarose gel electrophoresis.

#### 2.8.3 Reverse transcription, preamplification, and high-throughput qPCR

The BioMark HD system (Standard BioTools, South San Francisco, CA, USA) was used for high-throughput qPCR, following a protocol described in our previous study (Akram et al., 2024c, 2025). cDNA synthesis was performed using RT MasterMix (Standard BioTools, South San Francisco, CA, USA). Preamplification was conducted in a 96-well qPCR plate with a primer mixture and PreAmp Mastermix (Standard BioTools, South San Francisco, CA, USA) under the following thermal conditions: 95 °C for 2 min, then 14 cycles of 95 °C for 15 s and 60 °C for 4 min. Exonuclease I treatment was then applied to remove unincorporated primers, and pre-amplified cDNA samples were diluted 8-fold. Prior to high-throughput qPCR, we prepared a sample mixture by combining 2.25 μL of exonuclease-treated, pre-amplified cDNA with 2.5 μL of 2 × SSoFast EvaGreen Supermix (BioRad, Hercules, CA, USA) and 0.25 μL of 20 × DNA-binding dye (Standard BioTools). The assay mixture contained 0.5 μL of each primer (100 μmol/L), 2.5 μL of 2 × Assay Loading Reagent (Standard BioTools), and 2.25 μL of low-EDTA DNA suspension buffer (TEKnova, Hollister, CA, USA). Both sample and assay mixtures were loaded into 96.96 Integrated Fluid Circuits (IFCs), and qPCR was conducted with initial denaturation at 95 °C for 60 s, followed by 30 cycles of 96 °C for 5 s and 60 °C for 20 s. The standard curve, generated from dilutions of the pooled pre-amplified cDNA, was used to calculate relative mRNA levels. Four reference genes (*TBP*, *B2M*, *NDUFA*, and β-actin) were identified as the most stable under the experimental conditions using the NormFinder algorithm (Andersen et al., 2004). Relative gene expression was calculated using the Pfaffl method (Pfaffl, 2001) through normalization of target genes to the geometric mean of the reference genes' expression levels. Genes showing poor amplification efficiency, high quantification cycle (Cq) variability between technical replicates, or mean Cq values above 30 were excluded from downstream statistical analyses to ensure reliability of the expression data.

### 2.9 DNA extraction, 16S rRNA gene amplicon sequencing and bioinformatics

Cecal digesta DNA was extracted using the QIAamp PowerFecal Pro DNA Kit (Qiagen Benelux B.V., Venlo, Netherlands) following the manufacturer’s protocol. DNA concentration and quality were assessed following the same protocol as described in the RNA extraction section. For sequencing library preparation, the V3–V4 region of the 16S rRNA gene was amplified using primers 341F (5′-CCTAYGGGRBGCASCAG-3′) and 806R (5′-GGACTACNNGGGTATCTAAT-3′), each with sample-specific barcodes. Libraries were sequenced on an Illumina NovaSeq 6000 platform, which produced 250 bp paired-end reads. Ultrapure water was included as a negative control to monitor sequencing quality.

The raw sequences were subjected to quality filtering, trimming, and demultiplexing in QIIME2 (v2024.2) with the default settings. Low-quality reads were removed, and amplicon sequence variants (ASVs) were generated using DADA2. Taxonomic assignment of ASVs was conducted with the Naïve Bayes classifier against the SILVA database (release 138) at a 99% similarity threshold. For statistical analysis, the QIIME2 artifacts were imported into R (v4.2.3, R Foundation, Vienna, Austria). Microbial diversity metrics (Shannon and Simpson indices) were calculated as alpha diversity measures in R using the phyloseq package (v1.40.0) after rarefaction to the minimum sample depth, and groups were compared via the Kruskal–Wallis test. Beta diversity was evaluated with Bray–Curtis dissimilarity and visualized through principal coordinate analysis (PCoA). Group differences were assessed using non-parametric permutational multivariate analysis of variance (PERMANOVA) with 9999 permutations (vegan package, v2.6.4). Differential microbial abundance at the genus level was determined using linear discriminant analysis effect size (LEfSe) using the microbiome package (v1.18.0), with a linear discriminant analysis (LDA) score threshold of ≥2.0 and significance set at *P* < 0.05. False discovery rate (FDR) adjustment was applied using the Benjamini-Hochberg method (Benjamini and Hochberg, 1995), and the results were visualized as log_10_ (LDA score) values.

### 2.10 Statistical analysis

Data normality was assessed using the Shapiro–Wilk test in R before conducting statistical analyses. Outliers were identified and removed based on values exceeding quartile 3 + 1.5 × interquartile range or falling below quartile 1 − 1.5 × interquartile range. Growth performance, digestive organ characteristics, ileal histomorphology, intestinal permeability, and ileum gene expression were analyzed using a one-way ANOVA model as follows:

$$Y_{\text{ij}}=\mu +T_{i}+\epsilon_{\text{ij}},$$

where *Y*_*ij*_ is the dependent variable; *μ* is the overall mean; *T*_*i*_ is the fixed treatment effect; *ϵ*_*ij*_ is the residual error term.

Pairwise comparisons were conducted using Tukey’s HSD test. *P* < 0.05 was set significant difference and 0.05 < *P* ≤ 0.10 as a tendency. For gene expression data, *P*-values were adjusted for FDR using the Benjamini–Hochberg method. All the results are reported as the means with a pooled standard deviation (SD), which combines the variability observed in all samples. Principal component analysis (PCA) was performed to visualize sample clustering on the basis of gene expression data using the factoextra package (v1.0.7) in R. The multivariate effects of the treatments on sample clustering were tested using PERMANOVA with the adonis2 function (v2.6.4) to test for multivariate effects of dietary treatments on sample clustering in PCA. Heatmaps were plotted to show the sample variability and gene expression levels using the pheatmap package (v1.0.12) in R. Two-way hierarchical clustering of the heatmap data was performed using Pearson’s correlation distance and Ward’s clustering methods, with gene expression levels scaled per gene.

## 3 Results

### 3.1 Growth performance

On d 7 and 16, the BW of the HBWC group was higher than that of all LBW groups (*P* < 0.05; Table 3). Among LBW groups on d 16, the LBW + OH group had higher BW than the LBWC group (*P* < 0.001), LBW + CC chicks and LBW + CO chicks had intermediate weights. By d 38, the HBWC group maintained the highest BW, exceeding the LBWC group (*P* < 0.001). LBW + OH birds showed a significantly reduced difference in BW compared to HBWC birds and had significantly higher BW than LBWC birds (*P* < 0.001), while LBW + CO and LBW + CC birds showed intermediate values.

**Table 3:** Effects of coarse corn and oat hulls on the growth performance of low-weight broilers^1^.

Item	Day	Groups					SD	*P-*value
		HBWC	LBWC	LBW + CC	LBW + OH	LBW + CO		
BW, g	7	203.3^a^	164.5^b^	166.6^b^	165.9^b^	164.5^b^	16.45	<0.001
	16	675.8^a^	583.9^c^	598.5^bc^	611.7^b^	602.3^bc^	61.12	<0.001
	38	3087^a^	2796^b^	2928^ab^	2983^a^	2941^ab^	105.4	<0.001
ADG, g/d	8 to 16	52.5^a^	46.6^c^	47.6^bc^	49.5^b^	48.6^b^	2.45	<0.001
	17 to 38	109.6	100.6	105.9	107.9	106.5	5.36	0.086
	8 to 38	80.1^a^	72.4^c^	76.1^b^	77.3^ab^	76.3^b^	4.31	0.001
ADFI, g/d	8 to 16	71.7^a^	56.2^b^	57.6^b^	58.1^b^	56.8^b^	9.98	0.023
	17 to 38	146.1	134.7	142.7	140.0	137.4	7.40	0.062
	8 to 38	124.5^a^	111.9^b^	118.0^ab^	116.2^ab^	114.0^b^	6.60	0.005
FCR, g/g	8 to 16	1.22	1.20	1.20	1.19	1.17	0.161	0.735
	17 to 38	1.33	1.33	1.33	1.31	1.29	0.069	0.850
	8 to 38	1.34	1.31	1.31	1.29	1.27	0.070	0.608

Values with different superscripts in a row differ at *P* < 0.05.

HBWC = high BW chickens fed a commercial broiler diet with 10% finely ground corn; LBWC = low body weight chickens fed a commercial broiler diet with 10% finely ground corn; LBW + CC = low body weight chickens fed a commercial broiler diet with 7% coarse corn and 3% finely ground corn; LBW + OH = low body weight chickens fed a commercial broiler diet with 10% ground corn and 3% oat hulls; LBW + CO = low body weight chickens fed a commercial broiler diet with 7% coarse corn and 3% oat hulls. BW = body weight; ADG = average daily gain; ADFI = average daily feed intake; FCR = feed conversion ratio; SD = standard deviation.

1 The BW was measured individually using the animal as the experimental unit. The ADG ADFI and FCR were calculated from 6 replicates of each group using the pen as an experimental unit.

From d 8 to 16, the HBWC group had higher ADG than all other groups (*P* < 0.001). Among the LBW groups, the LBW + OH group exhibited greater ADG than the LBWC group (*P* < 0.001). Over the full study period (d 8–38), HBWC birds maintained the highest ADG, significantly surpassing LBWC, LBW + CC, and LBW + CO birds (*P* = 0.001). The LBW + OH group attained the highest ADG among all LBW groups, significantly exceeding the LBWC group (*P* = 0.001).

From d 8 to 16, the HBWC group had higher ADFI than all LBW groups (*P* = 0.023). Over the entire study period, HBWC birds had the highest ADFI, significantly surpassing LBWC and LBW + CO birds (*P* = 0.005). LBW + CC and LBW + OH birds showed intermediate ADFI values that did not differ significantly from HBWC or LBWC birds.

### 3.2 Relative lengths and weights of digestive organs

On d 21, the LBW + OH group had the highest relative pancreas weight, exceeding that of the HBWC group (*P* = 0.005, Table 4). By d 38, the LBW + CO group showed a lower relative liver weight compared to the HBWC group (*P* = 0.027), whereas the other groups had liver weights comparable to HBWC birds. The gizzard relative weight was higher in LBW + CC, LBW + OH, and LBW + CO birds than in both HBWC and LBWC birds on d 21 and 38 (*P* < 0.05, respectively).

**Table 4:** Effects of coarse corn and oat hulls on the relative weights (g/100 g BW) and lengths (cm/100 g BW) of the digestive organs of low-weight broilers.

Item	Day	Groups					SD	*P*-value
		HBWC	LBWC	LBW + CC	LBW + OH	LBW + CO		
Pancreas, g	14	0.38	0.35	0.39	0.39	0.35	0.097	0.733
	21	0.29^b^	0.34^ab^	0.33^ab^	0.37^a^	0.33^ab^	0.045	0.005
	38	0.16	0.17	0.18	0.16	0.17	0.040	0.745
Liver, g	14	2.89	3.06	3.05	2.91	2.86	0.393	0.703
	21	2.72	2.77	2.87	2.78	2.79	0.265	0.758
	38	2.51^a^	2.35^ab^	2.34^ab^	2.38^ab^	2.21^b^	0.023	0.027
Gizzard, g	14	2.05	1.89	2.01	2.09	1.97	0.223	0.220
	21	1.60^b^	1.55^b^	1.78^a^	1.83^a^	1.82^a^	0.259	0.016
	38	0.90^b^	0.93^b^	1.01^a^	1.05^a^	0.99^a^	0.191	0.044
Small intestine, g	14	7.97	7.90	7.80	7.45	7.15	0.907	0.137
	21	11.49	7.62	7.22	6.98	6.98	6.475	0.374
	38	5.18	4.92	5.36	4.69	5.21	0.748	0.215
Cecum, g	14	0.72	0.83	0.78	0.70	0.78	0.207	0.560
	21	0.84	0.89	0.79	0.92	0.91	0.228	0.627
	38	0.69	0.66	0.75	0.71	0.76	0.191	0.688
Small intestine, cm	14	23.69	25.93	25.71	24.70	23.69	3.173	0.089
	21	15.33	15.81	16.36	15.99	15.94	1.387	0.339
	38	6.82	7.17	7.35	7.01	7.55	0.871	0.273
Cecum, cm	14	3.85	4.37	5.01	4.04	4.02	0.506	0.160
	21	2.64	2.89	2.85	2.91	2.86	0.342	0.291
	38	1.27	1.46	1.38	1.42	1.50	0.230	0.143

HBWC = high BW chickens fed a commercial broiler diet with 10% finely ground corn; LBWC = low body weight chickens fed a commercial broiler diet with 10% finely ground corn; LBW + CC = low body weight chickens fed a commercial broiler diet with 7% coarse corn and 3% finely ground corn; LBW + OH = low body weight chickens fed a commercial broiler diet with 10% ground corn and 3% oat hulls; LBW + CO = low body weight chickens fed a commercial broiler diet with 7% coarse corn and 3% oat hulls. SD = standard deviation.

Values with different superscripts in a row indicate a significant difference (*n* = 12, *P* < 0.05).

### 3.3 Ileal histomorphology

On d 21, HBWC birds had greater VH compared to LBWC and LBW + CC birds (*P* = 0.005, Table 5). VH in LBW + OH and LBW + CO birds was intermediate, significantly exceeding LBW + CC (*P* = 0.005) but comparable to HBWC birds. By d 38, LBW + OH birds showed the highest VH, significantly surpassing LBWC birds (*P* = 0.042). On d 14, CD was lowest in LBW + CC compared to LBWC, LBW + OH, and LBW + CO birds (*P* = 0.017).

**Table 5:** Effects of coarse corn and oat hulls on the villus height, crypt depth and villus height to crypt depth of low-weight broilers.

Item	Day	Groups					SD	*P-*value
		HBWC	LBWC	LBW + CC	LBW + OH	LBW + CO		
Villus height, μm	14	548.8	541.3	525.3	542.5	544.9	53.90	0.863
	21	704.9^a^	633.9^bc^	605.8^c^	663.1^ab^	653.3^abc^	68.35	0.005
	38	955.5^ab^	881.8^b^	929.1^ab^	998.9^a^	951.8^ab^	95.77	0.042
Crypt depth, μm	14	148.0^ab^	161.2^a^	142.0^b^	163.6^a^	163.9^a^	20.73	0.017
	21	168.9	164.4	157.2	161.4	158.6	19.42	0.608
	38	139.6	131.4	140.1	132.9	139.6	21.26	0.769
Villus height/crypt depth ratio	14	3.72	3.40	3.76	3.36	3.36	0.517	0.123
	21	4.26	3.88	3.87	4.14	4.18	0.557	0.284
	38	6.90	6.82	6.79	7.64	6.90	1.008	0.201

HBWC = high BW chickens fed a commercial broiler diet with 10% finely ground corn; LBWC = low body weight chickens fed a commercial broiler diet with 10% finely ground corn; LBW + CC = low body weight chickens fed a commercial broiler diet with 7% coarse corn and 3% finely ground corn; LBW + OH = low body weight chickens fed a commercial broiler diet with 10% ground corn and 3% oat hulls; LBW + CO = low body weight chickens fed a commercial broiler diet with 7% coarse corn and 3% oat hulls. SD = standard deviation.

Values with different superscripts in a row indicate a significant difference (*n* = 12, *P* < 0.05).

### 3.4 Intestinal permeability

On d 14, 21, and 38, absolute plasma FITC-dextran concentrations did not differ significantly among groups (*P* > 0.05, Table 6). On d 14, relative plasma FITC-dextran levels were lower in the LBW + CC, LBW + OH, and LBW + CO groups compared to the LBWC group (*P* = 0.014) and were comparable to the HBWC group. On d 38, LBWC birds had a significantly higher relative plasma FITC-dextran concentration than HBWC birds (*P* = 0.022), while LBW groups fed CC, OH or their combination had intermediate values that were not significantly different from those of the HBWC group.

**Table 6:** Effects of coarse corn and oat hulls on plasma absolute and relative fluorescein isothiocyanate (FITC) dextran levels in low-weight broilers.

Item	Day	Groups					SD	*P-*value
		HBWC	LBWC	LBW + CC	LBW + OH	LBW + CO		
Absolute plasma FITC-dextran, ng/mL	14	45.7	55.8	46.1	48.2	48.3	10.49	0.119
	21	52.2	53.0	51.1	51.4	51.9	4.95	0.931
	38	59.7	64.8	64.0	62.1	63.1	5.45	0.429
Relative plasma FITC-dextran, ng/mL per 100 g BW	14	7.27^b^	10.05^a^	8.17^b^	8.29^b^	8.03^b^	2.027	0.014
	21	4.23	4.98	4.72	4.65	4.75	0.593	0.063
	38	1.88^b^	2.31^a^	2.12^ab^	2.03^ab^	2.08^ab^	0.283	0.022

HBWC = high BW chickens fed commercial broiler feed with fine corn; LBWC = low body weight chickens fed a commercial broiler diet with 10% finely ground corn; LBW + CC = low body weight chickens fed a commercial broiler diet with 7% coarse corn and 3% finely ground corn; LBW + OH = low body weight chickens fed a commercial broiler diet with 10% ground corn and 3% oat hulls; LBW + CO = low body weight chickens fed a commercial broiler diet with 7% coarse corn and 3% oat hulls. SD = standard deviation.

Values with different superscripts in a row indicate a significant difference (*n* = 12, *P* < 0.05).

### 3.5 Cecal VFA composition

Isobutyrate tended to be lower in the LBW + CO group on d 14 (*P* = 0.091, Table 7). On d 21, valerate concentration was highest in the LBW + CC group, significantly exceeding that of the LBW + OH group (*P* = 0.002). Isobutyrate, isovalerate and total BCFAs on the same day were significantly lower in the LBW + OH group compared to the LBWC group (*P* < 0.05, respectively).

**Table 7:** Effects of coarse corn and oat hulls on cecal volatile fatty acids (mmol/g wet digesta) in low-weight broilers.

Item	Day	Groups					SD	*P-*value
		HBWC	LBWC	LBW + CC	LBW + OH	LBW + CO		
Acetate	14	250.0	226.8	225.7	265.6	210.4	81.34	0.368
	21	246.8	259.1	245.3	255.8	243.1	65.38	0.978
	38	244.0	320.0	252.3	277.9	278.3	106.87	0.477
Propionate	14	24.8	20.5	21.4	22.6	20.8	3.75	0.125
	21	23.5	25.3	23.6	22.2	21.2	6.23	0.468
	38	27.6	31.8	29.8	31.3	27.6	8.98	0.704
Butyrate	14	59.8	48.1	43.1	61.2	44.1	20.33	0.111
	21	59.5	56.6	45.5	57.5	53.2	22.59	0.644
	38	64.9	81.0	69.2	71.4	75.1	39.03	0.695
Valerate	14	3.3	3.2	3.3	3.6	3.2	0.55	0.564
	21	3.41^a^	3.34^ab^	3.54^a^	3.04^b^	3.26^ab^	0.423	0.002
	38	4.24	4.26	4.69	4.39	4.02	1.103	0.535
Total SCFAs	14	320.9	290.5	291.1	352.9	271.9	107.34	0.314
	21	333.2	344.3	317.9	338.5	320.6	85.18	0.912
	38	340.7	437.1	356.0	385.1	385.0	146.49	0.436
Isobutyrate	14	2.7	3.6	2.9	2.6	2.5	0.94	0.091
	21	2.57^ab^	2.93^a^	2.78^a^	2.31^b^	2.51^ab^	0.351	0.005
	38	3.71	3.26	3.83	3.68	3.91	0.986	0.564
Isovalerate	14	2.3	2.8	2.7	2.7	2.4	0.91	0.392
	21	2.31^ab^	2.63^a^	2.41^ab^	2.03^b^	2.24^ab^	0.325	0.003
	38	3.41	2.91	3.57	3.29	3.42	0.913	0.479
Total BCFAs	14	4.9	6.1	5.1	5.1	4.9	1.71	0.491
	21	4.89^ab^	5.56^a^	4.94^ab^	4.34^b^	4.76^ab^	0.751	0.029
	38	7.12	6.17	7.40	6.97	7.33	1.867	0.496

HBWC = high BW chickens fed commercial broiler feed with fine corn; LBWC = low body weight chickens fed a commercial broiler diet with 10% finely ground corn; LBW + CC = low body weight chickens fed a commercial broiler diet with 7% coarse corn and 3% finely ground corn; LBW + OH = low body weight chickens fed a commercial broiler diet with 10% ground corn and 3% oat hulls; LBW + CO = low body weight chickens fed a commercial broiler diet with 7% coarse corn and 3% oat hulls. SD = standard deviation; BCFAs = branched-chain fatty acids.

Values with different superscripts in a row indicate a significant difference (*n* = 12, *P* < 0.05).

### 3.6 Ileal gene expression

#### 3.6.1 Principal component analysis and heatmap clustering

The PCA on d 14 and 38 showed no distinct clustering of experimental groups (Fig. S1). This was supported by PERMANOVA, which revealed no significant associations between principal component variability and gene expression differences on d 38, though a tendency for differentiation was observed on d 14 (*P* = 0.073). Heatmaps visualized gene expression variability across samples from all groups (Figs. S2 and S3). Consistent with PCA, 2-way hierarchical clustering on d 14 and 38 demonstrated that neither samples nor genes grouped consistently by experimental group or functional category.

#### 3.6.2 Differential gene expression

To identify differentially expressed genes, one-way ANOVA with Tukey’s HSD post hoc test was performed, considering genes with an FDR-adjusted *P* < 0.05 as significant (Table 8).

**Table 8:** Effects of coarse corn and oat hulls on the relative expression of genes involved in various intestinal functions in the ileum on d 14 and 38^1^.

Genes	Function	Groups					SD	FDR-adjusted *P*-value
		HBWC	LBWC	LBW + CC	LBW + OH	LBW + CO		
Day 14								
*CLDN1*	Barrier function	1.34^b^	1.37^b^	1.10^b^	2.26^a^	1.42^b^	0.827	0.004
*CLDN4*	Barrier function	0.54^b^	0.98^ab^	0.51^b^	1.62^a^	1.57^a^	1.084	0.003
*CLDN5*	Barrier function	1.58^ab^	0.86^bc^	0.45^c^	1.89^a^	0.74^bc^	1.019	0.001
*CDX*	Barrier function	0.98	1.05	0.99	1.33	1.39	0.440	0.083
*CCK*	Gut hormone	1.72^ab^	2.33^a^	1.13^b^	0.97^b^	1.73^ab^	1.052	0.021
*T1R1*	Nutrient receptor	0.89^b^	1.09^b^	0.92^b^	1.70^a^	0.95^b^	0.618	0.011
*NOS2*	Immune function	0.87^b^	0.83^b^	1.03^b^	1.50^a^	1.11^ab^	0.588	0.044
*TLR4*	Immune function	0.56^b^	0.96^ab^	0.52^b^	1.14^a^	0.67^b^	0.758	0.021
*IL-6*	Immune function	0.24^b^	0.22^b^	0.90^a^	0.32^b^	0.56^ab^	0.535	0.017
*SLC15A1*	Nutrient transport	0.64^c^	0.62^c^	1.63^ab^	2.07^a^	1.23^bc^	0.881	<0.001
*SLC1A4*	Nutrient transport	1.00^b^	1.02^b^	0.88^b^	1.94^a^	1.26^ab^	0.752	0.008
*SLC2A1*	Nutrient transport	0.98	0.85	0.98	1.70	1.45	0.816	0.064
*VDR*	Nutrient transport	1.39	1.20	1.01	1.51	0.59	0.894	0.085
*CALB1*	Nutrient transport	1.60	1.09	1.20	1.14	1.09	0.500	0.081
*HMOX2*	Oxidation	0.71^b^	0.78^b^	0.94^ab^	1.17^a^	0.94^ab^	0.397	0.045
Day 38								
*CLDN1*	Barrier function	0.94^a^	0.51^ab^	0.33^b^	0.69^ab^	0.79^ab^	0.613	0.027
*CLDN2*	Barrier function	1.06	1.23	2.02	1.57	1.39	0.859	0.079
*IL-18*	Immune function	1.40^a^	1.09^ab^	0.39^c^	0.50^bc^	0.58^bc^	0.695	0.003
*TLR4*	Immune function	0.42	0.96	0.27	0.46	0.36	0.641	0.094
*SLC15A1*	Nutrient transport	0.78^b^	1.05^b^	2.97^a^	1.34^b^	1.06^b^	0.903	<0.001
*SLC2A2*	Nutrient transport	1.63^a^	0.91^b^	0.76^b^	0.42^b^	0.51^b^	0.515	0.008
*SLC34A2*	Nutrient transport	0.45^b^	0.89^ab^	1.03^ab^	1.31^a^	0.95^ab^	0.814	0.049
*SLC30A1*	Nutrient transport	1.59	1.52	2.17	1.94	2.22	0.764	0.084

HBWC = high BW chickens fed a commercial broiler diet with 10% finely ground corn; LBWC = low body weight chickens fed a commercial broiler diet with 10% finely ground corn; LBW + CC = low body weight chickens fed a commercial broiler diet with 7% coarse corn and 3% finely ground corn; LBW + OH = low body weight chickens fed a commercial broiler diet with 10% ground corn and 3% oat hulls; LBW + CO = low body weight chickens fed a commercial broiler diet with 7% coarse corn and 3% oat hulls. SD = standard deviation; FDR = false discovery rate.

Values with different superscripts in a row indicate a significant difference (*n* = 12, FDR adjusted *P* < 0.05).

1 Only significantly different or tended to be different genes are shown.

On d 14, the LBW + OH group showed the highest expression of the tight junction genes *CLDN1*, *CLDN4*, and *CLDN5; CLDN1* was even significantly higher than that of the LBWC and HBWC groups (*P* = 0.004), while *CLDN4* expression was significantly higher compared to the HBWC group (*P* = 0.003) and *CLDN5* was significantly higher compared to the LBWC group (*P* = 0.001). The gut hormone *CCK* gene was upregulated in LBWC birds compared to LBW + CC and LBW + OH birds (*P* = 0.021), with intermediate levels in HBWC and LBW + CO birds. Expression of the nutrient receptor *T1R1* was significantly higher in LBW + OH birds than in all other groups (*P* = 0.011). Among the immune function genes, *TLR4* and *NOS2* (pattern recognition receptor and anti-inflammatory marker, respectively) showed the highest expression in LBW + OH birds (*P* < 0.05, respectively), although *TLR4* was not significantly different from LBWC birds, and *NOS2* was not significantly different from LBW + CO birds. The inflammatory marker *IL-6* was significantly upregulated in LBW + CC birds relative to HBWC, LBWC, and LBW + OH birds (*P* = 0.017). Genes associated with nutrient transport, including *SLC15A1* (peptide transporter) and *SLC1A4* (amino acid transporter), were upregulated in LBW + OH birds compared with LBWC and HBWC birds (*P* < 0.001 and *P* = 0.008, respectively). Additionally, the expression of genes such as *SLC2A1* (glucose transporter) and *VDR* (vitamin D receptor) tended to increase in LBW + OH birds (*P* > 0.05), whereas the expression of *CALB1* (calcium-binding protein) tended to increase in HBWC birds (*P* = 0.081). The expression of *HMOX2*, a gene associated with intestinal oxidation, was increased in the LBW + OH group (*P* = 0.045).

On d 38, *CLDN1* expression was significantly upregulated in HBWC birds compared to LBW + CC birds (*P* = 0.027). *CLDN2* expression tended to be higher in LBW + CC birds (*P* = 0.079). The expression of the proinflammatory cytokine *IL-18*, an immune-related gene, was highest in HBWC birds, significantly exceeding LBW + CC, LBW + OH and LBW + CO groups (*P* = 0.003). Among nutrient transporters, *SLC15A1* (peptide transporter) was significantly upregulated in LBW + CC birds (*P* < 0.001), whereas *SLC2A2* (glucose transporter) was highest in HBWC birds (*P* = 0.008). *SLC34A2* expression (sodium-phosphate cotransporter) was significantly upregulated in LBW + OH compared to HBWC (*P* = 0.049), while *SLC30A1* (zinc transporter) showed a tendency toward higher expression in the LBW + CO group (*P* = 0.084).

### 3.7 Cecal microbiota

Cecal amplicon sequencing generated 15,130,747 reads, with 86,461 ± 8181 (mean ± SD) reads per sample. After quality filtering, 13,617,672 reads remained, with an average of 77,815 ± 7363 reads per sample.

#### 3.7.1 Core microbiota composition

Compositional analysis revealed considerable inter-individual variability and a significant shift in the gut microbiota from d 14 to d 38 post-hatching. On d 14, the Firmicutes phylum dominated (95%–97%), with minor contributions from Bacteroidota (1%–2%), Proteobacteria (1%–3%), and Cyanobacteria (0.2%–1.8%) (Table S2). By d 21, Firmicutes remained predominant (93%–96%), but there were slight increases in Actinobacteriota (0.2%–3.6%). On d 38, the dominance of Firmicutes persisted (92%–95%), while Actinobacteriota (1.8%–2.8%) and Cyanobacteria (0.6%–1.2%) remained stable. Additionally, Bacteroidota exhibited minor fluctuations (1%–2%) over time, and low-abundance phyla such as Desulfobacterota (<0.3%) remained consistently present.

**Fig. 1:**
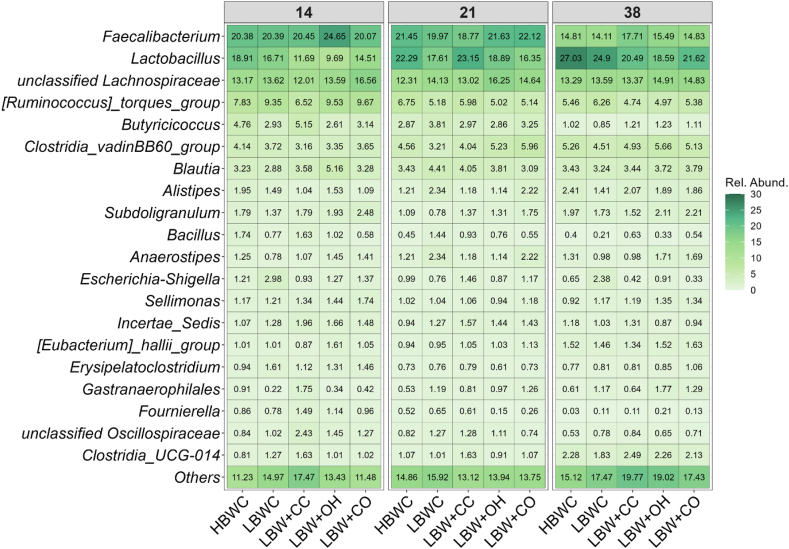
Relative abundance (%) of cecal bacterial genera in broilers from different dietary groups (*n* = 12). Groups include high body weight broilers fed a commercial broiler diet with 10% finely ground corn (HBWC), low body weight chickens fed a commercial broiler diet with 10% finely ground corn (LBWC), low body weight chickens fed a commercial broiler diet with 7% coarse corn and 3% finely ground corn (LBW + CC), low body weight chickens fed a commercial broiler diet with 10% finely ground corn and 3% oat hulls (LBW + OH), and low body weight chickens fed a commercial broiler diet with 7% coarse corn and 3% oat hulls (LBW + CO). The values indicate the mean relative abundance of bacterial genera.

At the genus level, core genera including *Faecalibacterium*, *Lactobacillus*, *unclassified Lachnospiraceae,* and *Ruminococcus torques group* accounted for approximately 56% of the total relative abundance in the chickens, as shown in Fig. 1. With age, *Lactobacillus* and *unclassified Lachnospiraceae* numerically increased, whereas *Faecalibacterium* and *Ruminococcus torques group* decreased. Despite the consistency of these core genera across individuals, many low-abundance genera collectively made up more than 10% of the community, representing a highly variable component of the gut ecosystem.

#### 3.7.2 Alpha and beta diversity

**Fig. 2:**
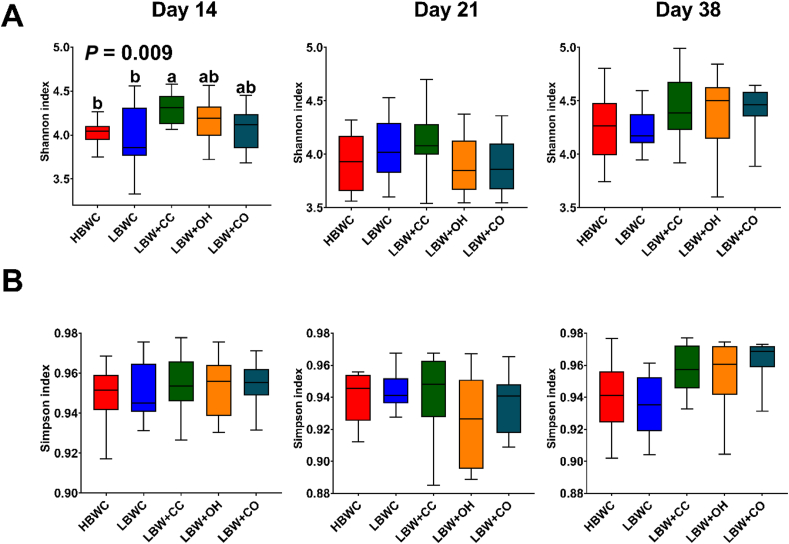
Shannon (A) and Simpson (B) indices of alpha diversity for the cecal microbiota in broilers from different dietary groups (*n* = 12). Groups include high body weight broilers fed a commercial broiler diet with 10% finely ground corn (HBWC), low body weight chickens fed a commercial broiler diet with 10% finely ground corn (LBWC), low body weight chickens fed a commercial broiler diet with 7% coarse corn and 3% finely ground corn (LBW + CC), low body weight chickens fed a commercial broiler diet with 10% finely ground corn and 3% oat hulls (LBW + OH), and low body weight chickens fed a commercial broiler diet with 7% coarse corn and 3% oat hulls (LBW + CO). Alpha diversity measures were evaluated using Kruskal–Wallis test, with significance threshold set at *P* < 0.05. Values in a row with no common letters differ significantly (*P* < 0.05).

On d 14, the Shannon index of alpha diversity was significantly higher in the LBW + CC group compared to LBWC and HBWC groups (*P* < 0.05, Fig. 2), with the LBW + OH and LBW + CO groups showing intermediate values. No significant differences in alpha diversity metrics were observed among groups on d 21 and 38. Beta diversity analysis using Bray–Curtis dissimilarity did not reveal a clear separation in the overall microbiota composition between groups at any timepoint (Fig. S4). This lack of separation was confirmed by PERMANOVA, which found no significant associations between principal component variability and microbiota composition differences (*P* > 0.05).

#### 3.7.3 Differential abundance analysis of bacterial genera

LEfSe analysis identified differentially abundant bacterial genera across groups on d 14, 21, and 38 (LDA cut-off ≥2.0, FDR-adjusted *P* < 0.05).

**Fig. 3:**
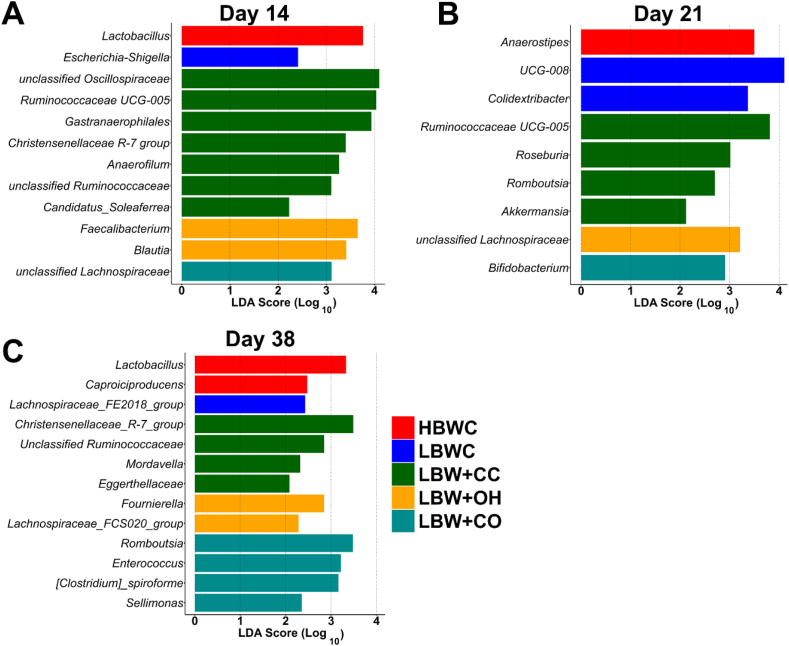
Linear discriminant analysis effect size (LEfSe) results of differentially abundant cecal bacterial genera in broilers from different dietary groups on d 14 (A), d 21 (B), and d 38 (C) (*n* = 12). Groups include high body weight broilers fed a commercial broiler diet with 10% finely ground corn (HBWC), low body weight chickens fed a commercial broiler diet with 10% finely ground corn (LBWC), low body weight chickens fed a commercial broiler diet with 7% coarse corn and 3% finely ground corn (LBW + CC), low body weight chickens fed a commercial broiler diet with 10% finely ground corn and 3% oat hulls (LBW + OH), and low body weight chickens fed a commercial broiler diet with 7% coarse corn and 3% oat hulls (LBW + CO). Only genera with a false discovery rate (FDR) adjusted *P* ≤ 0.05 and an absolute value of linear discriminant analysis (LDA) ≥ 2.0 are presented.

On d 14, a total of 12 genera were identified across groups (Fig. 3A). The HBWC group was enriched with *Lactobacillus*, whereas LBWC birds had increased *Escherichia-Shigella*. The LBW + CC group was enriched with *unclassified Oscillospiraceae*, *Ruminococcaceae UCG-005*, *Gastranaerophilales*, the *Christensenellaceae R-7 group*, *Anaerofilum*, *unclassified Ruminococcaceae,* and *Candidatus_Soleaferrea*. The LBW + OH group showed a higher abundance of *Faecalibacterium* and *Blautia*, whereas the LBW + CO group had higher abundance of *unclassified Lachnospiraceae*.

On d 21, a total of 9 genera were identified (Fig. 3B). HBWC birds were enriched with *Anaerostipes*, and LBWC birds with *UCG-008* (family Butyricicoccaceae) and *Colidextribacter*. LBW + CC birds showed higher abundance of *Ruminococcaceae UCG-005*, *Roseburia*, *Romboutsia,* and *Akkermansia*, while LBW + OH birds were enriched with *unclassified Lachnospiraceae*. LBW + CO birds showed increased *Bifidobacterium* abundance.

On d 38, a total of 13 genera were identified (Fig. 3C). HBWC birds showed a higher abundance of *Lactobacillus* and *Caproiciproducens*, while LBWC birds were enriched with *Lachnospiraceae_FE2018_group*. LBW + CC birds showed enrichment of *Christensenellaceae R-7 group* and *unclassified Ruminococcaceae*, *Mordavella*, and *Eggerthellaceae*. LBW + OH birds were enriched with *Fournierella* and *Lachnospiraceae_FCS020_group*, whereas LBW + CO birds showed increased abundance of *Romboutsia*, *Enterococcus*, *[Clostridium]_sprioforme* and *Sellimonas*.

## 4 Discussion

The relationship between first-week chick weight and subsequent broiler performance represents a critical determinant of production efficiency, as demonstrated by current findings and supported by previous research (Akram et al., 2024b). Despite uniform rearing conditions, HBWC chicks consistently outperformed LBWC chicks, with no compensatory growth observed in LBWC broilers throughout the experimental period. The better performance of HBWC birds corresponded with a higher ADFI, better intestinal function and favorable microbiota composition, corroborating earlier findings that heavier chicks possess intrinsic growth potential advantages over lighter chicks under standard conditions (Akram et al., 2024b,c). In LBWC chicks, higher expression of *CCK*, a gut hormone involved in satiety signaling and feed intake regulation (Everaert et al., 2019), may explain their lower feed intake. Furthermore, the dominance of *Lactobacillus*, a genus linked to probiotic effects and improved broiler growth (Zheng et al., 2019), in HBWC birds contrasts with the higher abundance of potential pathogen *Escherichia-Shigella* in LBWC chicks on d 14. The presence of this pathogenic genus, known to contribute to intestinal dysbiosis and inflammation (Akram et al., 2024b), likely contributes to the suboptimal performance of LBWC birds.

Dietary interventions with CC and OH, either individually or in combination, improved growth parameters in LBW broilers compared to the LBWC birds. By d 38, notably, the LBW + OH group not only outperformed the LBWC group but also showed a markedly reduced difference in BW compared to the HBWC group, primarily through increased feed intake rather than improved FCR. These findings suggest that OH inclusion modulates the feeding pattern, contributing to compensatory weight gain in LBW broilers. These results align with González-Alvarado et al. (2007), who reported increased weight gain and feed intake with 3% soybean or oat hulls, and Kheravii et al., 2018b, Kheravii et al., 2018a, who reported synergistic effects of CC and 2% sugar bagasse on broiler performance. The improved growth response of LBW broilers fed dietary interventions are attributed to enhanced GIT development and function. Dietary CC, OH or their combination rapidly increased gizzard size in LBW broilers, a response driven by increased mechanical demands for particle size reduction. The enlarged gizzard improves gut motility and digestive efficiency, partially mediated by CCK, which increases digesta retention time and pancreatic enzyme secretion, enhancing the interaction between digesta and digestive enzymes for better nutrient digestion (Amerah et al., 2007; González-Alvarado et al., 2008; Svihus, 2011). Increased transit time in the gizzard also lowers pH, stimulating pepsin activity (Dhakal, 2022) and accelerating protein denaturation, thereby improving protein digestion. Interestingly, ileal CCK expression was not significantly altered by dietary modifications, supporting findings that CCK may play a less central role in regulating pancreatic enzyme secretion in birds compared to mammals (Murai et al., 2000). The LBW + OH group exhibited increased relative pancreas weight, reflecting adjustments for increased enzyme production necessary for fibrous diet digestion. Previous research has demonstrated that insoluble fibers increase the production of pancreatic enzymes, including chymotrypsin (Bogułsawska-Tryk, 2005; Yokhana et al., 2016). Although enzyme activity and nutrient digestibility were not directly measured in this study, the results align with previous findings showing that structural diets promote gizzard development, thereby enhancing pancreatic enzyme secretion, nutrient digestibility and growth performance in broilers (Rasool et al., 2023; Yan et al., 2022). Dietary OH inclusion improved VH in LBW birds, counteracting the reduction in absorptive surface area observed in LBWC broilers. This aligns with findings by Moradi et al. (2021), who reported that OH stimulates mucosal development, which leads to improved intestinal morphology and nutrient absorption. At the molecular level, OH in LBW birds induced nutrient-sensing receptor *T1R1* upregulation, enhancing their capacity to detect and respond to essential nutrients including amino acids and fatty acids (Cordero et al., 2023). This was accompanied by the upregulation of nutrient transporters (*SLC15A1*, *SLC1A4*, and *SLC2A1*) on d 14, facilitating di- and tri-peptide, neutral amino acids, and glucose absorption. Additionally, *VDR*, which mediates vitamin D uptake, showed a tendency towards increased expression in the LBW + OH group, suggesting improved mineral utilization. These adaptations might have enabled LBW + OH birds to compensate for their initial growth disadvantage. The LBW + CC group also showed upregulation of the di- and tri-peptide transporter *SLC1A4* on d 38, indicating that CC can increase nutrient absorption during later growth stages. Nevertheless, its contribution to compensatory growth was less pronounced than that observed with OH. The structural properties of insoluble fibers, forming a bulky and spongy matrix, increase the interaction of digestive enzymes with digesta (Sarikhan et al., 2010). This enhances nutrient availability for enzymatic activity, improving nutrient absorption and retention, and supporting better growth performance. The higher nutrient availability in the intestinal lumen of LBW + OH birds likely activated nutrient transporters, consistent with Kheravii et al., 2018b, Kheravii et al., 2018a, who found dietary insoluble fiber (2% sugar bagasse) upregulated amino acid transporters in the small intestine. Additionally, OH stimulates pancreatic amylase secretion (Hetland et al., 2003), leading to increased carbohydrate digestion and glucose absorption.

The LBWC group exhibited increased intestinal permeability on both d 14 and 38 than HBWC group, as indicated by increased plasma FITC-dextran concentrations. This finding aligns with Akram et al. (2024c), who associated low BW in broilers with impaired gut barrier function and increased inflammation, facilitating pathogen translocation and elevating metabolic demands. Dietary OH reduced intestinal permeability, likely via upregulated expression of tight junction proteins *CLDN1*, *CLDN4*, and *CLDN5*, enhancing gut barrier integrity in LBW + OH broilers. This aligns with previous studies suggesting that insoluble fibers improve intestinal barrier function and host immunity by promoting SCFA production (Kheravii et al., 2018b). Unlike a previous study (Liebl et al., 2022), this study found a significant effect of OH on certain immune function genes. The observed upregulation of *NOS2* and *TLR4* in the LBW + OH group on d 14 suggests enhanced activation of innate immune pathways. *NOS2*, which is critical for nitric oxide production, contributes to pathogen clearance by promoting antimicrobial activity (Bogdan et al., 2000), whereas *TLR4* is integral for recognizing microbial components and activating downstream inflammatory signaling pathways, such as *NF-κB*, which regulates cytokine production (Molteni et al., 2016). The LBW + OH group demonstrated increased expression of *HMOX2*, a gene involved in antioxidant processes in the ileum, suggesting their increased capacity to mitigate free radicals in the intestine to maintain intestinal homeostasis (Akram et al., 2024c). These molecular alterations point to the effects of OH as a fermentable substrate for the gut microbiota, leading to the production of SCFAs that may influence *NOS2*- and *TLR4*-mediated pathways.

Dietary interventions showed minimal effects on overall SCFA concentrations but significantly influenced valerate levels, which were highest in LBW + CC broilers on d 21. Valerate has been associated with improved broiler performance and reduced necrotic enteritis (Onrust et al., 2018). Branched-chain fatty acids, primarily derived from protein fermentation in the cecum, are associated with undesirable microbial shifts and elevated nitrogenous metabolites (Qaisrani et al., 2015). The reduced BCFAs in the LBW + OH group on d 21 suggest that OH improved protein digestion in the small intestine, limiting cecal protein fermentation and production of microbial metabolites associated with gut dysbiosis, as corroborated by a previous study in weaner pigs (Kim et al., 2008).

Microbiota diversity, as indicated by the Shannon index, was greater in the LBW + CC group than in other groups on d 14, suggesting that CC feeding had a positive influence on microbial richness during early growth. No significant differences in alpha diversity were observed among CC- or OH-fed groups at later time points, which is consistent with the findings of previous studies (Adewole et al., 2021; Yan et al., 2022). In the LBW + CC group, the enrichment of the *Christensenellaceae R-7 group* and *Romboutsia* suggested their roles in enhancing nutrient metabolism. The *Christensenellaceae R-7 group* is a biomarker for broiler growth and muscle development (Akram et al., 2024b), while *Romboutsia*, a butyrate producer, supports intestinal health and energy availability, contributing to better feed efficiency (Chen and Yu, 2021). Meanwhile, OH promoted *Faecalibacterium* and *Blautia* abundance suggested their involvement in modulating gut architecture, SCFA production, and energy metabolism. *Faecalibacterium*, particularly *F. prausnitzii*, is a key butyrate producer known to reduce inflammation, strengthen intestinal barrier integrity and improvements in morphological traits (Fujimoto et al., 2013; Lopez-Siles et al., 2017), as reflected by reduced intestinal permeability, upregulated tight junction gene expression and increased VH in the LBW + OH group. *Blautia*, known for fermenting insoluble fibers such as those in OH, produces acetate, a key SCFA that promotes gut health and increases energy metabolism (Zhou et al., 2021). This may indicate that the use of OH as a substrate significantly influences the number of favorable bacteria. In the LBW + CO group, *unclassified Lachnospiraceae* were enriched on d 14 and their abundance in LBW + OH broilers on d 21 indicated their central role in fiber degradation and SCFA production, particularly butyrate. Butyrate is known to promote intestinal health and modulate immune function (Biddle et al., 2013).

The particle size of coarsely ground corn in this study did not seem to be the same as that of particles generated after milling when they were subjected to secondary grinding. Secondary grinding, which involved expander conditioning and pelleting, further reduced the particle size in CC and CO diets, substantially minimizing differences with control diet. Despite this reduction, the CC and CO diets positively influenced gizzard development and broiler performance. These results align with the findings of Ebbing et al. (2022), where secondary grinding using an expander and pelleting significantly reduced the initial particle size obtained after milling but enhanced nutrient digestibility. This outcome suggests that, beyond particle size alone, other physical characteristics such as pellet hardness and durability may also play a role in stimulating gizzard activity. Harder pellets that resist disintegration during early digestion can increase the mechanical function of the gizzard and prolong digesta retention, potentially improving feed intake and nutrient utilization (Abdollahi and Ravindran, 2013). However, pellet hardness and durability were not directly assessed in this study, which limits our ability to determine their specific contribution to the observed effects. Future studies should consider incorporating direct measurements of pellet physical quality to better understand how structural features interact with particle size in influencing digestive function. Maintaining coarse particles in broiler pellets is challenging, especially in high-throughput feed mills using conditioning with expander. The intense mechanical and thermal processes often break down larger particles into finer fractions. Balancing particle preservation with pellet durability and process efficiency is critical, as coarse particles are nutritionally beneficial but prone to degradation during production.

While all structural dietary interventions enhanced gizzard development to a comparable extent, OH addition conferred superior growth benefits in LBW broilers relative to CC. This differential response may reflect the distinct functional properties of OH, particularly its high lignocellulosic content, which provides not only mechanical stimulation but also fermentable substrates that shape the gut microbial landscape. Insoluble fiber can act as a physical matrix, supporting bacterial colonization and enhancing fermentation, leading to higher SCFA production. This improved SCFA profile, combined with the mechanical action of insoluble fiber in enhancing digestive efficiency, contributes to better nutrient utilization and intestinal health, potentially giving OH an advantage over CC. Interestingly, the combination of CC and OH did not consistently outperform OH alone. The simultaneous inclusion of CC and OH may dilute the specific benefits of each component, as CC primarily enhances gizzard activity through its coarse particle size, while OH provides advantages by supporting gut microbial activity and SCFA production. These combined effects may alter the gut environment in ways that reduce the distinct benefits of OH’s fiber-driven modulation of gut health, suggesting complex relationships between corn particle size and dietary fiber that warrant further investigation.

## 5 Conclusion

LBWC birds exhibited persistent growth impairments, characterized by compromised intestinal morphology, elevated gut permeability, and dysbiotic microbial signatures compared to HBWC birds, despite uniform post–hatch management. Dietary inclusion of CC and OH, either individually or in combination, enhanced GIT development by increasing the relative gizzard weight and reducing gut permeability. Among these dietary strategies, OH inclusion had the most significant impact, effectively ameliorating growth depression in LBW broilers, outperforming the LBWC group, and reducing the difference in BW compared to HBWC birds. This was achieved through improved ileal morphology, upregulation of genes associated with gut barrier function, nutrient transport, and immune function. Additionally, OH contributed to better gut health by promoting favorable caecal microbiota and reducing BCFAs. Overall, incorporating structural components, particularly OH at moderate inclusion levels (3%), effectively addressed growth limitations in LBWC birds, aligning their performance with HBWC birds by slaughter age and presenting a promising approach for optimizing production efficiency.

## Credit Author Statement

**Muhammad Zeeshan Akram:** Validation, Methodology, Conceptualization, Formal analysis, Visualization, Data curation, Writing – original draft, Software, Investigation. **Ester Arévalo Sureda:** Investigation, Writing – review & editing, Data curation. **Matthias Corion:** Writing – review & editing, Software, Visualization. **Luke Comer:** Visualization, Writing – review & editing, Software. **Haoran Zhao:** Writing – review & editing, Software, Visualization. **Martine Schroyen:** Writing – review & editing, Methodology, Supervision. **Nadia Everaert:** Resources, Methodology, Conceptualization, Writing – review & editing, Project administration, Funding acquisition, Supervision.

## Availability of data and material

The 16S rRNA gene sequencing data can be accessed under Bioproject accession PRJNA1202002 at the NCBI website.

## Declaration of Competing Interest

We declare that we have no financial and personal relationships with other people or organizations that can inappropriately influence our work, and there is no professional or other personal interest of any nature or kind in any product, service and/or company that could be construed as influencing the content of this paper.
