# Supplementary material for: Effects of coarse corn or oat hulls on growth performance, intestinal health, and microbiota modulation in underperforming broilers
Source: Anim Nutr. 2025 Aug 6;23:153–66. doi: 10.1016/j.aninu.2025.04.012 (PMC12663492; doi:10.1016/j.aninu.2025.04.012)
Supplement: Multimedia component 1 [file mmc1.docx]

**Table S1.** Primers used for high-throughput qPCR and a brief description of their main functions^1^.

| Function | Gene | Full name | Description | Primers (5’ 🡪 3’) | Accession no | Reference |
| --- | --- | --- | --- | --- | --- | --- |
| Barrier function | *CLDN1* | Claudin-1 | Transmembrane protein of tight junction (TJ) | F: CTTCATCATTGCAGGTCTGTCAG R: AAATCTGGTGTTAACGGGTGTG | NM_001013611.2 | Criado-Mesas et al. (2021) |
|  | *CLDN2* | Claudin-2 | Transmembrane protein of TJ | F: ACTGCAGCTGCCCTCGGT  R: AAGCTTCACCCTGCTGCTGT | NM_001277622.1 | Akram et al. (2024) |
|  | *CLDN3* | Claudin-3 | Transmembrane protein of TJ | F: GCCAAGATCACCATCGTCTC  R: CACCAGCGGGTTGTAGAAAT | NM_204202.2 | Barekatain et al. (2019) |
|  | *CLDN4* | Claudin-4 | Transmembrane protein of TJ | F: CTGTGCCGGGACACTGAATG  R: TCCTCCACAGTGGTGTTTGG | XM_003642382.6 | Akram et al. (2024) |
|  | *CLDN5* | Claudin-5 | Transmembrane protein of TJ | F: GTCCCAGAAGCGGGAGATAG R: CGAGTACTTGACGGGGAAGG | NM_204201.2 | Akram et al. (2024) |
|  | *OCLN* | Occludin | Protein of TJ involved in both inter-membrane and paracellular diffusion of small molecules | F: ACGGCAGCACCTACCTCAA R: GGGCGAAGAAGCAGATGAG | NM_205128.1 | Criado-Mesas et al. (2021) |
|  | *ZO-1* | Zonula Occludens-1 | Scaffold proteins that form part of the cytoplasmic plaque of TJ | F: ACCACAAGGAGCCATTCCAG  R: GTGAGGCCCACACATTACCA | XM_040680624.2 | Criado-Mesas et al. (2021) |
|  | *ZO-2* | Zonula Occludens-2 | Scaffold proteins that form part of the cytoplasmic plaque of TJ | F: GCCCAGCAGATGGATTACTT  R: TGGCCACTTTTCCACTTTTC | XM_040655419.2 | Criado-Mesas et al. (2021) |
|  | *JAM-2* | Junctional Adhesion Molecule-2 | Transmembrane protein of TJ | F: GGTACTTGGGGGTCTTCTGC R: TGTGCTTGCAACTAAGAATAGCC | NM_001397141.1 | Criado-Mesas et al. (2021) |
|  | *JAM-3* | Junctional Adhesion Molecule-3 | Transmembrane protein of TJ | F: CCAGAGTGTTGAGCTGTCCT  R: AGAATTTCTGCCCGAGTTGC | XM_417876 | Criado-Mesas et al. (2021) |
|  | *MUC2* | Mucin-2 | Secretory mucine important in the establishment of the mucus layer | F: CCCTGGAAGTAGAGGTGACTG R: TGACAAGCCATTGAAGGACA | XM_046942297.1 | Criado-Mesas et al. (2021) |
|  | *MUC13* | Mucin-13 | Transmembrane mucine that plays a role in cell signalling pathways | F: CCAGGCACCAGAAGTGCTAA  R: TGCGTACTGATGCACGTAGT | XM_003641585.6 | Criado-Mesas et al. (2021) |
|  | *MUC5ac* | Mucin-5AC | Mucin 5ac | F: TGTGGTTGCTATGAGAATGGA  R: TTGCCATGGTTTGTGCAT | XM_003641322.2 | Forder et al. (2012) |
|  | *CDX* | Caudal Type Homeobox Transcription Factor | Intestinal tract development | F: ACAGCTGTCCCCTAATGCAC  R: TCCTTTGTCCTCGTCTTGCC | NM_204676.3 | Akram et al. (2024) |
| Gut hormone | *GHRL* | Ghrelin | Ghrelin: Hunger hormone. Induces motor activity in the intestinal tract | F: AACTGCTCTGGCTGGCTCT  R: CTCCCTCTGTTTCATCTGTAT | XM_046926185.1 | Criado-Mesas et al. (2021) |
|  | *Proglucagone* | Proglucagon | Precurose of GLP-1 | F: CACAAGGCACATTCACCAGT  R: TTCTTTGGCAGCTTGACCTT | NC_052538.1 | Herwig et al. (2020) |
|  | *Proglucagone B* | Proglucagon related protein | Precursor of GLP-1 | F: CACAAGGCACATTCACCAGT  R: TGGTATTCTCCCAAAAGGTCTC | NC_052538.1 | Herwig et al. (2020) |
|  | *PYY* | Peptide YY | Peptide tyrosine tyrosine. feed intake regulatory hormone | F: AGGAGATCGCGCAGTACTTCT  R: TGCTGCGCTTCCCATACC | NM_001361182.2 | Herwig et al. (2020) |
|  | *CCK* | Cholecystokinim | Feed intake regulatory hormone | F: GAAGGTAGGGAGCGGCAC  R: TCGGAAAAGGGGGAAAACGA | XM_015281332.4 | Song et al. (2012) |
| Nutrient receptors | *T1R1* | Taste Receptor Type 1 Member 1 | Taste receptor type 1 member 1 | F: GTGTCATCCCCACAACCAA  R: CACCACTGCCTCAAAGAAGG | XM_425734.4 | He et al. (2018) |
|  | *T1R3* | Taste Receptor Type 1 Member 3 | Taste receptor type 1 member 3 | F: CATTACCGTCTTCGCCACTC  R: CTCTGTTCAAATCGGGCTTC | XM_425740.3 | He et al. (2018) |
|  | *GRP43* | G-protein coupled receptor 43 | FFAR2 – activated by short chain fatty acids | F: AGGGAATCCGGGATGGAGAA  R: ACGCAGTCAGGTTGGTTCAA | NM_001318430.1 | Akram et al. (2024) |
|  | *GPR41* | G-protein coupled receptor 41 | FFAR3 – activated by short chain fatty acids | F: GAAGGTGGTTTGGGAGTGAA  R: CAGAGGATTTGAGGCTGGAG | XM_427629 | Zhang et al. (2021) |
|  | *GPR120* | G-protein coupled receptor 120 | FFAR4 – activated by medium-chain and unsaturated long-chain FFAs | F: ACTTCACTGCTTTGCCTCAGT  R: CCAGTACAAGTGGAGGGTTCA | XM_040675455.2 | Akram et al. (2024) |
| Immune response | *IL-4* | Interleukin-4 | Cytokine that induces differentiation of naive helper T cells (Th0 cells) to Th2 cells | F: TTATGCAAAGCCTCCACAATTG  R: GTGGGACATGGTGCCTTGAG | XM_046900385.1 | Criado-Mesas et al. (2021) |
|  | *IL-6* | Interleukin-6 | Humoral immunity related genes | F: CTCGTCCGGAACAACCTCAA  R: GGAGAGCTTCGTCAGGCATT | NM_204628.2 | Akram et al. (2024) |
|  | *IL-8* | Interleukin-8 | Secreted in response to pathogenic bacteria infection or specific inflammatory cytokines | F: AGATGTGAAGCTGACGCCAA  R: GAGCTGAGCCTTGGCCATAA | NM_205498.2 | Criado-Mesas et al. (2021) |
|  | *IL-10* | Interleukin-10 | Anti-inflammatory cytokine produced by activated macrophages and T cell | F: CTGAGGGTGAAGTTTGAGGAAAT  R: AGCCAAAGGTCCCCTTAAACTC | NM_001004414.4 | Criado-Mesas et al. (2021) |
|  | *IL-18* | Interleukin-18 | Pro-inflammatory cytokine. primarily produced by macrophages. targeting T helper type-1 (Th1) cells | F: CTCCTCCACACAGCAACACA  R: ATGCAGTTGGCCACTTCTGT | XM_046932259.1 | Criado-Mesas et al. (2021) |
|  | *IL-22* | Interleukin-22 | Commonly used as marker of inflammation involved in T-lymphocytes activation | F: ACATCAGGGAGAACAACCGC  R: TGCCACATCCTCAGCATACG | NM_001199614.1 | Criado-Mesas et al. (2021) |
|  | *IL-1β* | Interleukin-1β | Mediator of the inflammatory response and involved in cellular processes | F: CGCTACACCCGCTCACAGT  R: GCAATGTTGAGCCTCACTTTCTG | XM_046931582.1 | Criado-Mesas et al. (2021) |
|  | *TLR2* | Toll-like receptor 2 | Transmembrane receptor for the recognition of Gram positive bacteria | F: AGGCGATCCCAAGAGGTTC  R: TTTCCCAAAACATCTGCTGTTG | XM_046915412.1 | Criado-Mesas et al. (2021) |
|  | *TLR4* | Toll-like receptor 4 | Transmembrane receptor for the recognition of Gram negative bacteria | F: CAGTCCGTGCCTGGAGGT  R: TTGAGCTTAGCAATTTCAGACTGTTG | NM_001030693.2 | Criado-Mesas et al. (2021) |
|  | *TNF-α* | Tumor Necrosis Factor alpha | Regulation of the host immunity against multiple pathogens | F: TTGCGAGGGGAGAGGAGAAA  R: GTCAGTACCGCGTCGTCTTT | XM_046927261.1 | Criado-Mesas et al. (2021) |
|  | *AHSA1* | Activator of Hsp90 ATPase | Co-chaperone activator of HSP90 | F: GGGGAAGCCTCCATCAACAA  R: TCACTCCTGTGGTCGAGGT | XM_040672816.2 | Criado-Mesas et al. (2021) |
|  | *AvBD6* | Avian β-defensin 6 | Avian defense involved in antimicrobial functions and protecting the gut epithelium | F: CTTGCTGTGTGAGGAACAGGTG  R: TTTGGTAGTTGCAGGCAGGAT | NM_001001193.1 | Criado-Mesas et al. (2021) |
|  | *AvBD9* | Avian β-defensin 9 | Avian defense with antimicrobial properties and other cellular functions | F: CTGAGACCTCACTGACCACG  R: GTGCTCCCAGGACTCTTCAC | NM_001001611.3 | Criado-Mesas et al. (2021) |
|  | *HSPA4* | Heat Shock Protein Family A Member 4 | Member of HSP proteins and play a prominent role in repair and protection of the intestinal environment | F:TGAGACTAATAAATGAATCAACTGCAGT  R: CCCCATATCCACAAAAACAACA | XM_046927108.1 | Criado-Mesas et al. (2021) |
|  | *IFNG* | Interferon gamma | Host defense for combating against the intracellular pathogens including Salmonella | F: ACCTTCCTGATGGCGTGAAG  R: CTGAAGAGTTCATTCGCGGC | NM_205149.2 | Criado-Mesas et al. (2021) |
|  | *NOS2* | Nitric Oxide Synthase 2 | Induce the development of Th1 type of IR in infections | F: CTCCAGCAGAGCTTCTACC TCAA  R: GCCAGGTGCTCTTCTATTT TTAATTC | NM_204961.2 | Criado-Mesas et al. (2021) |
|  | *PTGES* | Prostaglandin E synthase | Intestinal inflammatory factor | F: GGCTCTGAGGACAATGCAGA  R: CCAGAGGAGAGCACAGCAAA | XM_046928949.1 | Criado-Mesas et al. (2021) |
|  | *Cox-1* | Cyclooxygenase-1 | Cyclo-oxygenase 1 | F: GCGCATCAGTAGACCTAGCC  R: TGGTATTGTGACAGTGCGGG | JX160009.1 | Konieczka et al. (2019) |
|  | *Cox-2* | Cyclooxygenase-2 | Cyclo-oxygenase 2 | F:ATTCCTGACCCACAAGGCAC  R: AGTCAACCCCATGGCCGTAA | NM_001167719 | Hollemans et al. (2020) |
|  | *Lox-12* | Lipoxygenase-12 | Lipo-oxygenase | F: CTGATTACGCCGTGCTGGAT  R: ATTGGGGCACACAGGAATGT | XM_015274997.1 | Konieczka et al. (2019) |
|  | *CYP450* | Cytochrome P450 | Cytochromes P450 | F: ACCACTTCTGGAAGGAGGGA  R: CGCTCTCGTAGACACCCAAC | D49803.1 | Konieczka et al. (2019) |
| Nutrient transport | *SLC15A1* | Solute Carrier Family 15 Member 1 | Peptide transporter-1 | F: CAGGATTTCCCTGTGTCAGGT  R: GCAGCGTGGACAAGTATGG | XM_046906441.1 | Criado-Mesas et al. (2021) |
|  | *SLC1A1* | Solute Carrier Family 1 Member 1 | Excitatory amino acid transporter | F: TGCTGCTTTGGATTCCAGTGT  R:AGCAATGACTGTAGTGCAGAAGTAATATATG | XM_046936555.1 | Criado-Mesas et al. (2021) |
|  | *SLC1A4* | Solute Carrier Family 1 Member 4 | Neutral amino acid transporter by ASC system | F: ACAGCAAGCTGTGGTCAGAA  R: TCTCCCAGAATGCAATCACAGT | XM_046914471.1 | Criado-Mesas et al. (2021) |
|  | *SLC3A1* | Solute Carrier Family 3 Member 1 | Protein related to neutral amino acid transporter | F: CCCGCCGTTCAACAAGAG  R: AATTAAATCCATCGACTCCTTTGC | XM_040667709.2 | Criado-Mesas et al. (2021) |
|  | *SLC7A9* | Solute Carrier Family 7 Member 9 | Na+-independent neutral/cysteine. cationic amino acid exchanger | F:CAGTAGTGAATTCTCTGAGTGTGAAGCT  R: GCAATGATTGCCACAACTACCA | XM_046925529.1 | Su et al. (2014) |
|  | *SLC6A19* | Solute Carrier Family 6 Member 19 | Na+-dependent neutral amino acid transporter | F: CCAGAGGGCAATGTAACCCA  R: AAGGCTAAGCCGGTTCCTTC | XM_040663289.2 | Akram et al. (2024) |
|  | *SLC7A1* | Solute Carrier Family 7 Member 1 | Transport lysine. arginine. and histidine | F: CAAGAGGAAAACTCCAGTAATTGCA  R: AAGTCGAAGAGGAAGGCCATAA | XM_046941902.1 | Su et al. (2014) |
|  | *SLC7A2* | Solute Carrier Family 7 Member 2 | Transport lysine. arginine. and histidine | F: TGCTCGCGTTCCCAAGA  R: GGCCCACAGTTCACCAACAG | XM_046916218.1 | Su et al. (2014) |
|  | *SLC7A5* | Solute Carrier Family 7 Member 5 | Transport hydrophobic amino acids | F: ACGTGCAAGCTCACACCTAA  R: CGAGGCCTCCTCAACTCTCA | NM_001030579.3 | Akram et al. (2024) |
|  | *SLC7A6* | Solute Carrier Family 7 Member 6 | Na+-dependent neutral/cationic amino acid exchanger | F: GCCCTGTCAGTAAATCAGACAAGA  R: TTCAGTTGCATTGTGTTTTGGTT | XM_040681080.2 | Su et al. (2014) |
|  | *SLC7A7* | Solute Carrier Family 7 Member 7 | L amino acid transporter 2 | F: CAGAAAACCTCAGAGCTCCCTTT  R: TGAGTACAGAGCCAGCGCAAT | XM_046911929.1 | Criado-Mesas et al. (2021) |
|  | *SLC2A1* | Solute Carrier Family 2 Member 1 | Glucose transporter-1 | F: GCAAGATGACAGCTCGCCT  R: GCTCCTCATATCGGTACAGCC | NM_205209.2 | Akram et al. (2024) |
|  | *SLC2A2* | Solute Carrier Family 2 Member 2 | Glucose transporter-2 | F: CAGGAACGTTGGTCCTCTCC  R: GCGCCCATAGTGTGCTTCTA | NM_207178.2 | Criado-Mesas et al. (2021) |
|  | *SLC5A1* | Solute Carrier Family 5 Member 1 | Sodium glucose transporter 1 | F: GCCATGGCCAGGGCTTA  R: CAATAACCTGATCTGTGCACCAGTA | XM_046928028.1 | Criado-Mesas et al. (2021) |
|  | *SLC2A5* | Solute Carrier Family 2 Member 5 | Transport fructose | F: AAAGAGCTGTAGGTGTGGGC  R: CTTTTGCCTGGTTGCCTTCC | XM_040689119.2 | Akram et al. (2024) |
|  | *SLC5A9* | Solute Carrier Family 5 Member 9 | Sodium glucose transporter-4 | F: ATACCCAAGGTCATAGTCCCAAAC  R: TGGGTCCCTGAACAAATGAAA | XM_020589419.5 | Su et al. (2014) |
|  | *FABP* | Fatty Acid Binding Protein | Liver fatty acid binding protein | F: TGAATGTGGCTGGCTCGATTT  R: CAGGTTGACCCCTCCTGTACG | AY563636.1 | Akram et al. (2024) |
|  | *FABP1* | Fatty Acid Binding Protein 1 | Fatty acid binding protein | F: CATCTTCTCTTGTGTTGGGAGC  R: TGATCATCAGGAAGCCCGAG | NM_204192.4 | Akram et al. (2024) |
|  | *FABP2* | Fatty Acid Binding Protein 2 | Related with epithelial cell content and occurrence | F: ATGGAAGCAATGGGCGTGAA  R: TTCGATGTCGATGGTACGGA | NM_001007923.2 | Criado-Mesas et al. (2021) |
|  | *FABP6* | Fatty Acid Binding Protein 6 | Necessary for the transport of bile acids in the gut and it is associated with bacterial presence and inflammation | F: CGGTCTCCCTGCTGACAAGA  R: CCACCTCGGTGACTATTTTGC | XM_046926910.1 | Criado-Mesas et al. (2021) |
|  | *SLC34A2* | Solute Carrier Family 34 Member 2 | Intestinal phosphate absorption and phosphate homeostasis | F: TGGGGAGAAAGAAGTGTCACAGA  R: GTGAAGCCACGTTGCCTTTGT | NM_204474.3 | Criado-Mesas et al. (2021) |
|  | *VDR* | Vitamin D Receptor | Transcription factor that mediates the vitamin D3. involved in signalling intestinal calcium and phosphate absorption | F- GCAAAAGGCCGAGAAATGGG R- GAACACCCGTGGCAGATTCA | XM_046934191.1 | Criado-Mesas et al. (2021) |
|  | *ATP1A1* | ATPase Na+/K+ Transporting Subunit Alpha 1 | ATPase Na+/K+ transporting subunit alpha 1 (Calcium transporter) | F- TGCAAATCCATCAGAATCTCGT R- TCCTCATCCAAGGGTTGCAC | XM_046906976.1 | Akram et al. (2024) |
|  | *SLC30A1* | Solute Carrier Family 30 Member 1 | Efflux of Zn2+ | F: TGGGTGATATGAAGGAC  R: AACCTAAGGCATCTCCA | NM_001389457.2 | Akram et al. (2024) |
|  | *CALB1* | Calbindin 1 | Calcium transporter | F- GGCAGGCTTGGACTTAACACC R- GTCGGCAACACCTGAGCAAG | NM_205513.2 | Zanu et al. (2020) |
| Metabolism | *Cox-16* | Cytochrome c oxidase subunit 16 | Enzyme involved in the generation of energy by the mitochondria | F: CCTGCTTTGAAGGAAAAATTGAAG  R: CCAAGTCAGATTGTTCCAATTTCTC | NM_001197057.2 | Criado-Mesas et al. (2021) |
|  | *EIF4EBP1* | Eukaryotic Translation Initiation Factor 4E | mTOR pathway proteins—protein synthesis and cell proliferation | F: ATTGAGAACAACCATGTCCAGAAC  R: ATGTCAAACTGCTCTTCTTCACCT | XM_040689367.2 | Criado-Mesas et al. (2021) |
|  | *mTOR* | Mechanistic Target of Rapamycin | mTOR pathway proteins—protein synthesis and cell proliferation | F: TGCTGACAAACGCTATGGAGGT  R: AGCCATGACACTGTCCTTATGCT | XM_040689168.2 | Criado-Mesas et al. (2021) |
|  | *RPS6KB1* | Ribosomal Protein S6 Kinase B1 | mTOR pathway proteins—protein synthesis and cell proliferation | F: ACACCTGTTGATAGCCCAGATGA  R: GCCACATACGTAAAACCCAGAAA | XM_046930143.1 | Criado-Mesas et al. (2021) |
| Oxidation | *GPX7* | Glutathione Peroxidase 7 | Intracellular antioxidant. and plays a great role in the detoxification of various peroxides | F: GGTGCCTCCTTTCCTATGTTCA  R: GTTGGTTCTTCTCCAGTAGAATCAA | NM_001163245.2 | Criado-Mesas et al. (2021) |
|  | *HIF1A* | Hypoxia Inducible Factor 1 Alpha | Transcription factor that regulates genes involved in inflammation and cell death | F: CACTTTTTCAGGCAGTTGGAATTG  R: TTTTGCACGCCTTTACACGTT | XM_046917646.1 | Criado-Mesas et al. (2021) |
|  | *HMOX2* | Heme Oxygenase 2 | Oxidative stress marker | F: TCCAGTCCACGATGGGAAA  R: GCATTGCCTGCTAGCTTGTCT | XM_040684168.2 | Criado-Mesas et al. (2021) |
|  | *SOD1* | Superoxide Dismutase 1 | Antioxidant enzyme | F: CCGGCTTGTCTGATGGAGAT  R: CTGCGCTGGTACACCCATTT | NM_205064.2 | Criado-Mesas et al. (2021) |
|  | *XDH* | Xanthine Dehydrogenase | Enzyme associated to the synthesis of reactive oxygen species and is member of cellular defense system | F: GAAGCCATTCCATTACTTCAGTTATG  R: AATGTCTGTGCGGATGTTCTTG | XM_046913189.1 | Criado-Mesas et al. (2021) |
| Reference genes | *LBR* | Lamin B Receptor | Reference gene | F: CTAACCGTCGCTCAGGGC  F: TCCAAAAGCAATACCTGGCG | NM_001396139.1 | Criado-Mesas et al. (2021) |
|  | *NDUFA* | NADH  Oxidoreductase Subunit A | Reference gene | F: TGTGCAGAAACTACAGGACAAACTG  R: AGGGAAAGCTCATTTTCAGCCT | NM_001097637.1 | Criado-Mesas et al. (2021) |
|  | *YWHAZ* | Tyrosine 3-Monooxygenase/Tryptophan 5-Monooxygenase Activation Protein Zeta | Reference gene | F: GCAAGCAGAAAGCAAAGTTTTCT  R: TGTGATTGCTCCACAATCCCT | XM_046911632.1 | Criado-Mesas et al. (2021) |
|  | *GAPDH* | Glyceraldehyde-3-Phosphate Dehydrogenase | Reference gene | F: CGTGCAGCAGGAACACTA  R: CAGATCGATGAAGGGATC | NM_204305.2 | Akram et al. (2024) |
|  | *18S* | 18S Ribosomal RNA | Reference gene | F: ATTCCGATAACGAACGAGACT  R: GGACATCTAAGGGCATCACA | XR_006936397.1 | Chen et al. (2016) |
|  | *B-Actin* | Beta-Actin | Reference gene | F: TGACTGACCGCGTTACT R: GACCCACGATAGATGGGAA | NM_205518.2 | Akram et al. (2024) |
|  | *UB* | Ubiquitin | Reference gene | F: GGGATGCAGATCTTCGTGAAA R: CTTGCCAGCAAAGATCAACCTT | X02650.1 | Barekatain et al. (2019) |
|  | *RPS7* | Ribosomal Protein S7 | Reference gene | F: GGCGCTGAGCGAGAAAGG  R: CTCCAGGAGAGCCTGGGATA | XM_040667252.2 | This study |
|  | *B2M* | Beta-2-Microglobulin | Reference gene | F: TACTCCGACATGTCCTTCAACG  R: TCAGAACTCGGGATCCCACTT | NM_001001750.4 | Barekatain et al. (2019) |
|  | *GUSB* | Beta-Glucuronidase | Reference gene | F: GGCAGACTGGTCCTGTTGTTG  R: GGGTCCTGAGTGATGTCATTGA | NM_001039316.2 | Barekatain et al. (2019) |
|  | *TBP* | TATA-Box Binding Protein | Reference gene | F: AGCTCTGGGATAGTGCCACAG  R: ATAATAACAGCAGCAAAACGCTTG | XM_046913188.1 | Barekatain et al. (2019) |
|  | *TUBAT* | Tubulin Alpha | Reference gene | F: CAAGCATGAATGCCAACTCTCC  R: TCACGCATGGTTCGTCCT | NM_205444.2 | Akram et al. (2024) |
|  | *r28s* | 28S Ribosomal RNA | Reference gene | F: GGCGAAGCCAGAGGAAACT  R: GACGACCGATTTGCACGTC | XR_006936395.1 | Barekatain et al. (2019) |

^1^There were 79 target genes and 13 reference genes in total.

**Table S2**. Relative abundance (%) of phyla in caecal samples of broiler chickens on d 14, 21 and 38.

| Phylum | HBWC  (*n* = 12) | LBWC  (*n* = 12) | LBW+CC  (*n* = 12) | LBW+OH  (*n* = 12) | LBW+CO  (*n* = 12) | SD |
| --- | --- | --- | --- | --- | --- | --- |
| Day 14 |  |  |  |  |  |  |
| Firmicutes | 95.49 | 95.38 | 95.75 | 96.51 | 95.81 | 0.442 |
| Bacteroidota | 1.98 | 1.53 | 1.09 | 1.54 | 1.10 | 0.371 |
| Proteobacteria | 1.22 | 2.50 | 0.95 | 1.28 | 2.39 | 0.720 |
| Cyanobacteria | 0.90 | 0.20 | 1.75 | 0.34 | 0.42 | 0.634 |
| Actinobacteriota | 0.33 | 0.27 | 0.27 | 0.20 | 0.17 | 0.065 |
| Desulfobacterota | 0.08 | 0.11 | 0.18 | 0.13 | 0.10 | 0.037 |
| Other | 0.01 | 0.01 | 0.01 | 0.00 | 0.01 | 0.003 |
| Day 21 |  |  |  |  |  |  |
| Firmicutes | 94.29 | 93.56 | 95.90 | 94.87 | 91.59 | 1.618 |
| Bacteroidota | 1.23 | 2.44 | 1.20 | 1.18 | 2.23 | 0.624 |
| Proteobacteria | 1.14 | 1.11 | 1.68 | 1.00 | 1.22 | 0.265 |
| Cyanobacteria | 0.55 | 1.19 | 0.80 | 0.97 | 1.26 | 0.291 |
| Actinobacteriota | 2.62 | 1.46 | 0.22 | 1.84 | 3.60 | 1.269 |
| Desulfobacterota | 0.17 | 0.22 | 0.17 | 0.12 | 0.10 | 0.046 |
| Other | 0.01 | 0.03 | 0.03 | 0.02 | 0.00 | 0.012 |
| Day 38 |  |  |  |  |  |  |
| Firmicutes | 94.49 | 92.47 | 94.61 | 94.24 | 94.38 | 0.888 |
| Bacteroidota | 2.26 | 1.31 | 1.93 | 1.57 | 1.74 | 0.359 |
| Proteobacteria | 0.64 | 2.08 | 0.39 | 0.83 | 0.32 | 0.717 |
| Cyanobacteria | 0.57 | 1.08 | 0.61 | 1.03 | 1.17 | 0.282 |
| Actinobacteriota | 1.82 | 2.79 | 2.17 | 2.08 | 2.13 | 0.357 |
| Desulfobacterota | 0.21 | 0.26 | 0.27 | 0.23 | 0.23 | 0.023 |
| Other | 0.02 | 0.01 | 0.03 | 0.01 | 0.03 | 0.009 |

HBWC = high BW chickens fed a commercial broiler diet with 10% finely ground corn; LBWC = low body weight chickens fed a commercial broiler diet with 10% finely ground corn; LBW+CC = low body weight chickens fed a commercial broiler diet with 7% coarse corn and 3% finely ground corn; LBW+OH = low body weight chickens fed a commercial broiler diet with 10% ground corn and 3% oat hulls; LBW+CO = low body weight chickens fed a commercial broiler diet with 7% coarse corn and 3% oat hulls.


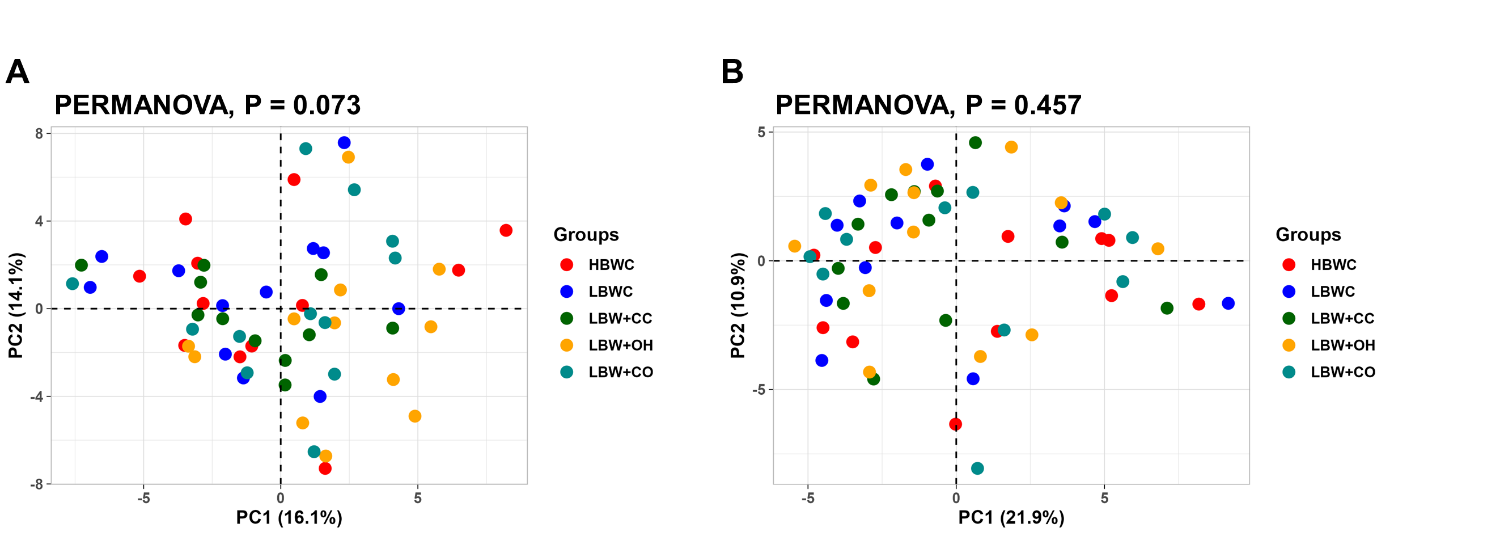
**Fig. S1**. Principal component analysis (PC1 and PC2) based on the gene expression in the ileum of high body weight broilers fed a commercial broiler diet with 10 % fine corn (HBWC, *n* = 12), low body weight chickens fed a commercial broiler diet with 10 % fine corn (LBWC, *n* = 12), and LBW broilers fed diets with 7% coarse corn (LBW+CC, *n* = 12), 3% oat hulls (LBW+OH, *n* = 12), or a CC+OH combination (LBW+CO, *n* = 12) on d 14 (A) and 38 (B). PERMANOVA = nonparametric permutational multivariate analysis of variance.

***P* = 0.457**

***P* = 0.073**


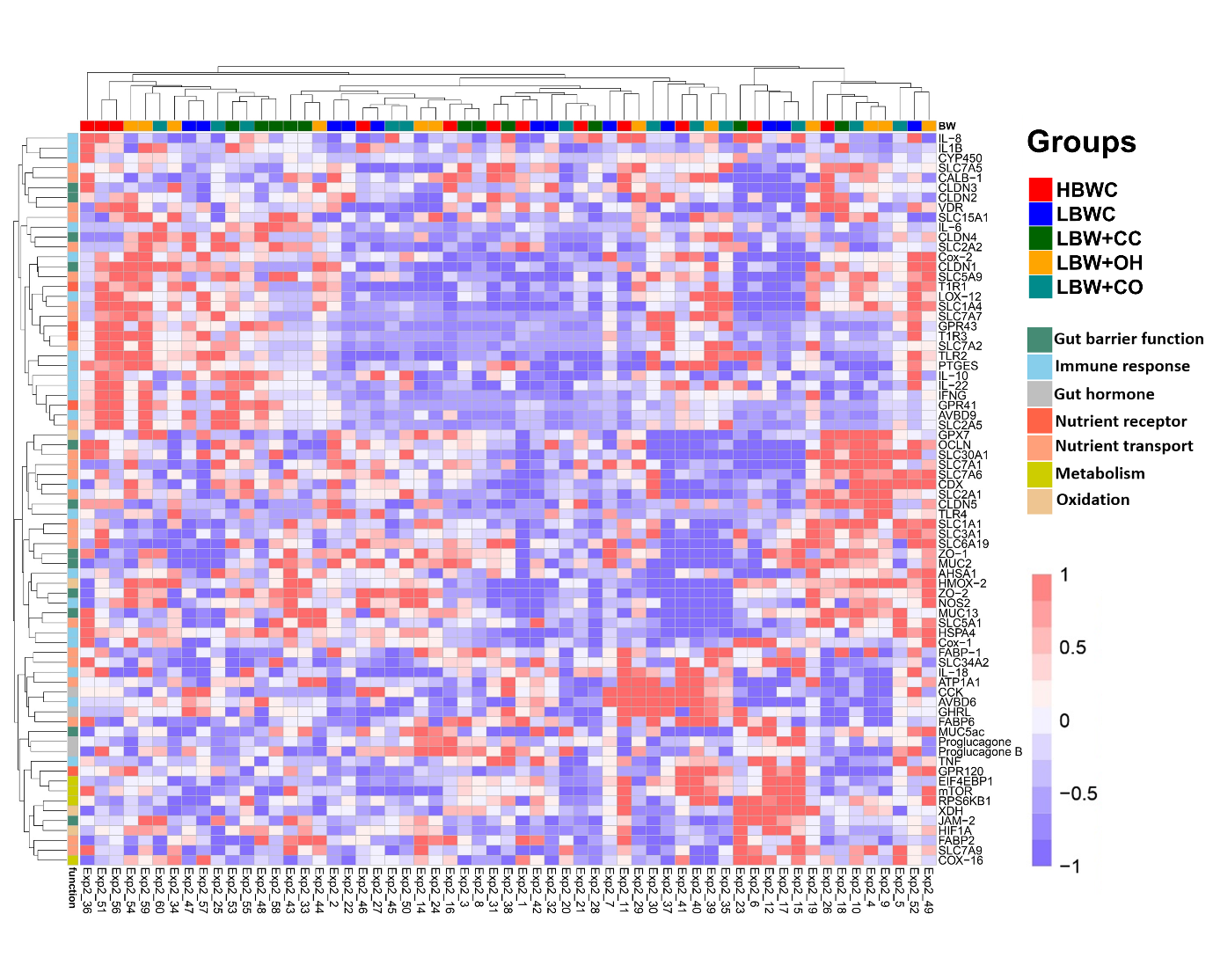
**Fig. S2**. Heatmap of ileal gene expression levels on d14 of high body weight broilers fed a commercial broiler diet with 10 % fine corn (HBWC, *n* = 12), low body weight chickens fed a commercial broiler diet with 10 % fine corn (LBWC, *n* = 12), and LBW broilers fed diets with 7% coarse corn (LBW+CC, *n* = 12), 3% oat hulls (LBW+OH, *n* = 12), or a CC+OH combination (LBW+CO, *n* = 12). The *x*-axis represents individual samples, while the *y*-axis shows the genes. Expression levels are color-coded, with red corresponding high expression and blue indicating low. Gene functions are denoted by different colors on the *y*-axis. The left dendrogram clusters genes with similar expression patterns, and the top dendrogram groups samples with similar gene expression profiles.


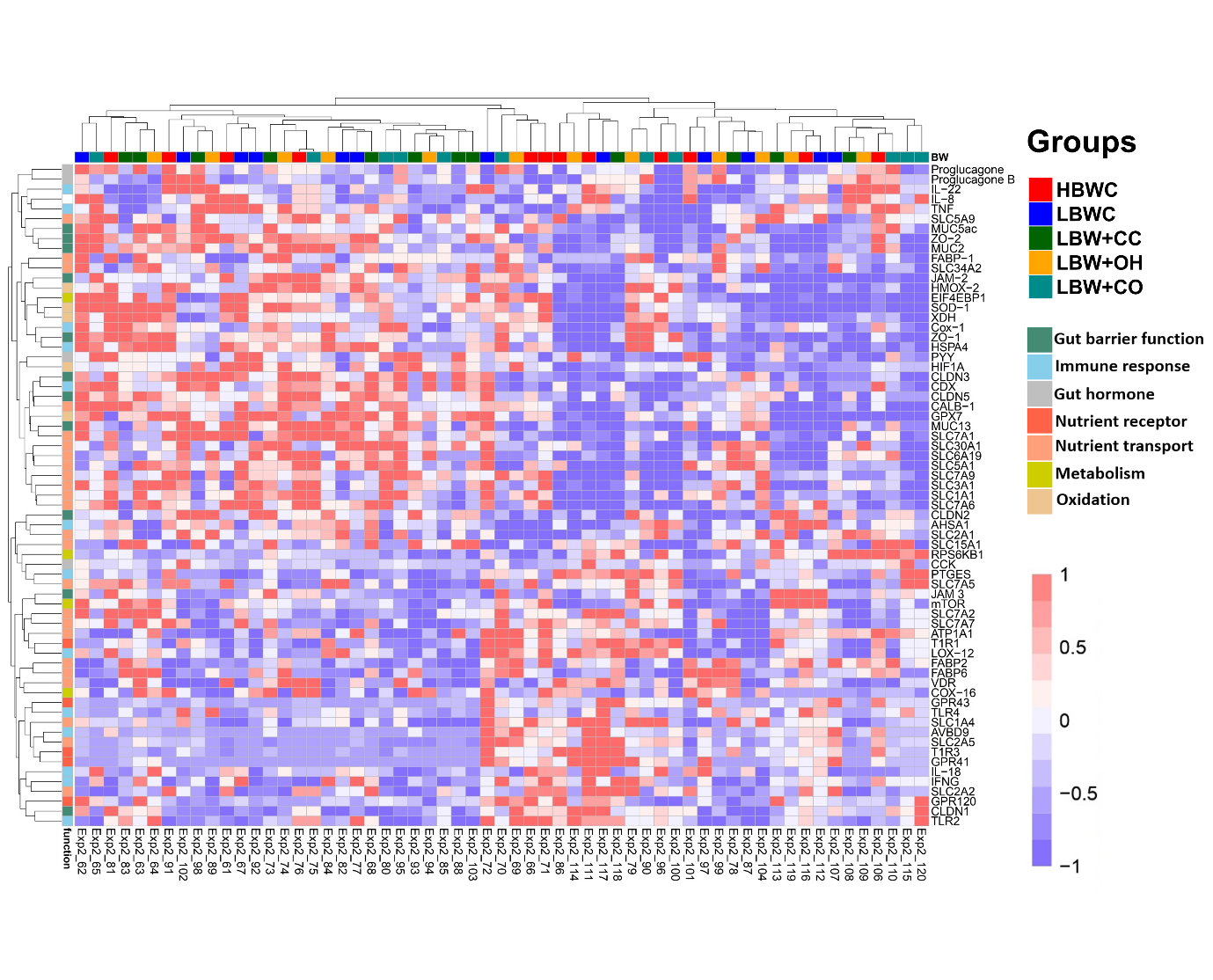


**Fig. S3**. Heatmap of ileal gene expression levels on d 38 of high body weight broilers fed a commercial broiler diet with 10 % fine corn (HBWC, *n* = 12), low body weight chickens fed a commercial broiler diet with 10 % fine corn (LBWC, *n* = 12), and LBW broilers fed diets with 7% coarse corn (LBW+CC, *n* = 12), 3% oat hulls (LBW+OH, *n* = 12), or a CC+OH combination (LBW+CO, *n* = 12). The x-axis represents individual samples, while the y-axis shows the genes. Expression levels are color-coded, with red corresponding high expression and blue indicating low. Gene functions are denoted by different colors on the y-axis. The left dendrogram clusters genes with similar expression patterns, and the top dendrogram groups samples with similar gene expression profiles.


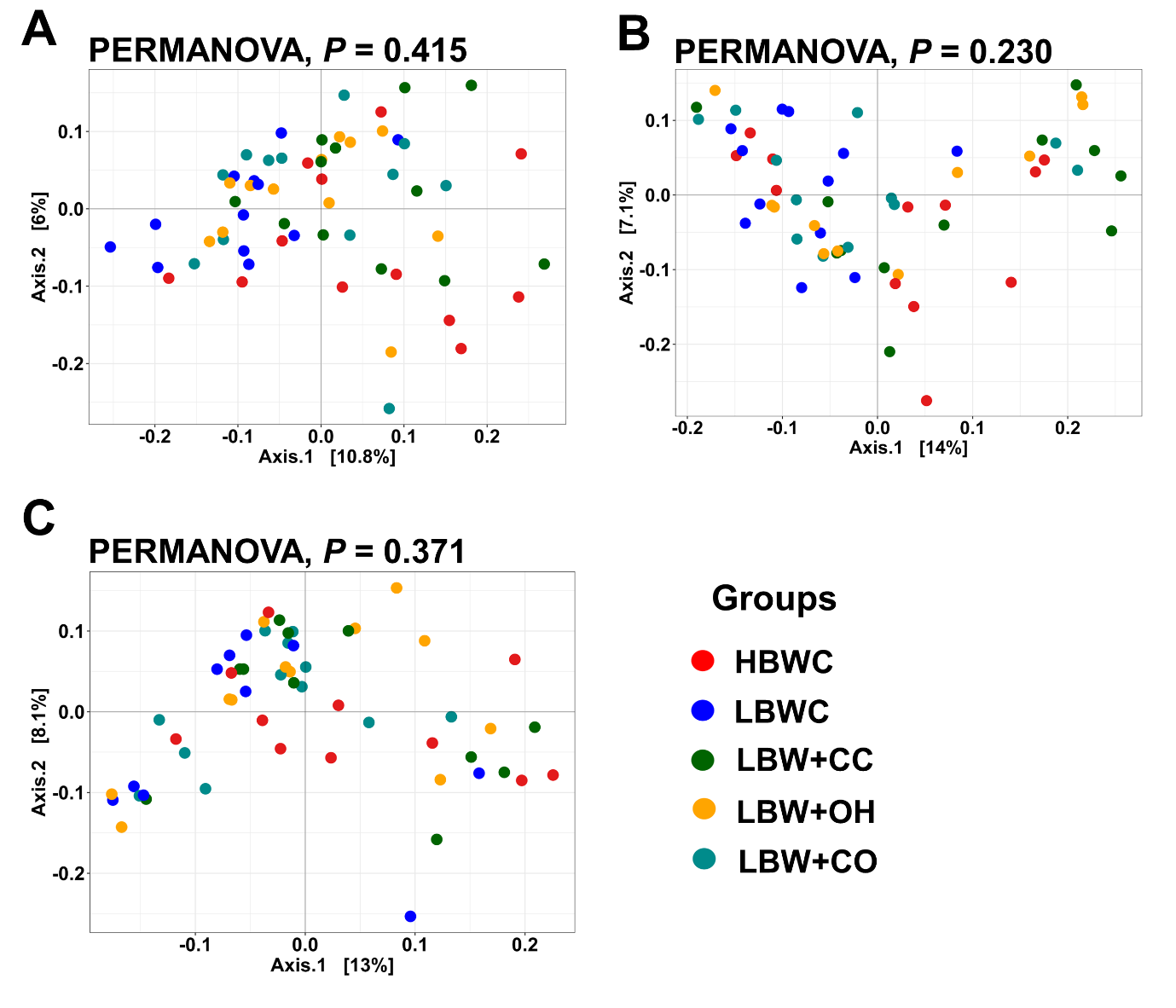


**Fig. S4.** Principal coordinate analysis (PCoA) for Bray–Curtis dissimilarity matrices of the cecal microbiota of high body weight broilers fed a commercial broiler diet with 10% fine corn (HBWC, *n* = 12), low body weight chickens fed a commercial broiler diet with 10% fine corn (LBWC, *n* = 12), and LBW broilers fed diets with 7% coarse corn (LBW+CC, *n* = 12), 3% oat hulls (LBW+OH, *n* = 12), or a CC+OH combination (LBW+CO, *n* = 12) on d 14 (A), d 21 (B), and d 38 (C). The multivariate effects of dietary treatments on β diversity were evaluated via nonparametric permutational multivariate analysis of variance (PERMANOVA), and significant differences were indicated as *P* < 0.05.

**References**

Barekatain, R., P. V Chrystal, G. S. Howarth, C. J. McLaughlan, S. Gilani, and G. S. Nattrass. 2019. Performance, intestinal permeability, and gene expression of selected tight junction proteins in broiler chickens fed reduced protein diets supplemented with arginine, glutamine, and glycine subjected to a leaky gut model. Poult. Sci. 98:6761–71.

Chen, X., K. Naehrer, and T. J. Applegate. 2016. Interactive effects of dietary protein concentration and aflatoxin B1 on performance, nutrient digestibility, and gut health in broiler chicks. Poult. Sci. 95:1312–25.

Criado-Mesas, L., N. Abdelli, A. Noce, M. Farré, J. F. Pérez, D. Solà-Oriol, R. Martin-Venegas, A. Forouzandeh, F. González-Solé, and J. M. Folch. 2021. Transversal gene expression panel to evaluate intestinal health in broiler chickens in different challenging conditions. Sci. Rep. 11:1–14.

Forder, R. E. A., G. S. Nattrass, M. S. Geier, R. J. Hughes, and P. I. Hynd. 2012. Quantitative analyses of genes associated with mucin synthesis of broiler chickens with induced necrotic enteritis. Poult. Sci. 91:1335–41.

He, X., Z. Lu, B. Ma, L. Zhang, J. Li, Y. Jiang, G. Zhou, and F. Gao. 2018. Effects of chronic heat exposure on growth performance, intestinal epithelial histology, appetite-related hormones and genes expression in broilers. J. Sci. Food Agric. 98:4471–8.

Herwig, E., K. Schwean-Lardner, A. Van Kessel, R. K. Savary, and H. L. Classen. 2020. Assessing the effect of starch digestion characteristics on ileal brake activation in broiler chickens. PLoS One 15:1–20.

Hollemans, M. S., J. van Baal, G. de Vries Reilingh, B. Kemp, A. Lammers, and S. de Vries. 2020. Intestinal epithelium integrity after delayed onset of nutrition in broiler chickens. Poult. Sci. 99:6818–27.

Konieczka, P., M. Barszcz, P. Kowalczyk, M. Szlis, and J. Jankowski. 2019. The potential of acetylsalicylic acid and vitamin e in modulating inflammatory cascades in chickens under lipopolysaccharide-induced inflammation. Vet. Res. 50:1–10.

Proszkowiec-Weglarz, M., L. L. Schreier, S. Kahl, K. B. Miska, B. Russell, and T. H. Elsasser. 2020. Effect of delayed feeding post-hatch on expression of tight junction– and gut barrier–related genes in the small intestine of broiler chickens during neonatal development. Poult. Sci. 99:4714–29.

Song, Z., L. Liu, A. Sheikhahmadi, H. Jiao, and H. Lin. 2012. Effect of Heat Exposure on Gene Expression of Feed Intake Regulatory Peptides in Laying Hens (D Fan, Ed.). J. Biomed. Biotechnol. 2012:484869.

Su, S., K. B. Miska, R. H. Fetterer, M. C. Jenkins, and E. A. Wong. 2014. Expression of digestive enzymes and nutrient transporters in Eimeria acervulina-challenged layers and broilers. Poult. Sci. 93:1217–26.

Zanu, H. K., S. K. Kheravii, N. K. Morgan, M. R. Bedford, and R. A. Swick. 2020. Interactive effect of dietary calcium and phytase on broilers challenged with subclinical necrotic enteritis: part 2. Gut permeability, phytate ester concentrations, jejunal gene expression, and intestinal morphology. Poult. Sci. 99:4914–28.

Zhang, J. ‐M., X. ‐Y. Liu, W. Gu, H. ‐Y. Xu, H. ‐C. Jiao, J. ‐P. Zhao, X. ‐J. Wang, H. ‐F. Li, and H. Lin. 2021. Different effects of probiotics and antibiotics on the composition of microbiota, SCFAs concentrations and FFAR2/3 mRNA expression in broiler chickens. J. Appl. Microbiol. 131:913–24.
